# Development, Characterization and Pharmacological Evaluation of Cannabidiol-Loaded Long Circulating Niosomes

**DOI:** 10.3390/pharmaceutics15102414

**Published:** 2023-10-03

**Authors:** Viliana Gugleva, Katerina Ahchiyska, Dilyana Georgieva, Rositsa Mihaylova, Spiro Konstantinov, Erik Dimitrov, Natalia Toncheva-Moncheva, Stanislav Rangelov, Aleksander Forys, Barbara Trzebicka, Denitsa Momekova

**Affiliations:** 1 Department of Pharmaceutical Technologies, Faculty of Pharmacy, Medical University of Varna, 84 Tsar Osvoboditel Str., 9000 Varna, Bulgaria; viliana.gugleva@mu-varna.bg; 2 Department of Pharmaceutical Technology and Biopharmaceutics, Faculty of Pharmacy, Medical University of Sofia, 2 Dunav Str., 1000 Sofia, Bulgaria; 105523@students.mu-sofia.bg (K.A.); dgeorgieva@pharmfac.mu-sofia.bg (D.G.); 3 Department of Pharmacology, Pharmacotherapy and Toxicology, Faculty of Pharmacy, Medical University of Sofia, 2 Dunav Str., 1000 Sofia, Bulgaria; rmihaylova@pharmfac.mu-sofia.bg (R.M.); skonstantinov@pharmfac.mu-sofia.bg (S.K.); 4 Institute of Polymers, Bulgarian Academy of Sciences, bl.103 Akad. G. Bonchev Str., 1113 Sofia, Bulgaria; e_dimitrov@polymer.bas.bg (E.D.); ntoncheva@polymer.bas.bg (N.T.-M.); rangelov@polymer.bas.bg (S.R.); 5 Centre of Polymer and Carbon Materials, Polish Academy of Sciences, ul. M. Curie-Skłodowskiej 34, 41-819 Zabrze, Poland; aforys@cmpw-pan.pl (A.F.); btrzebicka@cmpw-pan.pl (B.T.)

**Keywords:** star-shaped polyglycidol- poly(ε-caprolactone) copolymers, copolymer-modified niosomes, steric stabilization, EPR effect, cannabidiol, apoptosis, cytotoxicity

## Abstract

Cannabidiol (CBD) is a promising drug candidate with pleiotropic pharmacological activity, whose low aqueous solubility and unfavorable pharmacokinetics have presented obstacles to its full clinical implementation. The rational design of nanocarriers, including niosomes for CBD encapsulation, can provide a plausible approach to overcoming these limitations. The present study is focused on exploring the feasibility of copolymer-modified niosomes as platforms for systemic delivery of CBD. To confer steric stabilization, the niosomal membranes were grafted with newly synthesized amphiphilic linear or star-shaped 3- and 4-arm star-shaped copolymers based on polyglycidol (PG) and poly(ε-caprolactone) (PCL) blocks. The niosomes were prepared by film hydration method and were characterized by DLS, cryo-TEM, encapsulation efficacy, and in vitro release. Free and formulated cannabidiol were further investigated for cytotoxicity and pro-apoptotic and anti-inflammatory activities in vitro in three human tumor cell lines. The optimal formulation, based on Tween 60:Span60:Chol (3.5:3.5:3 molar ration) modified with 2.5 mol% star-shaped 3-arm copolymer, is characterized by a size of 235 nm, high encapsulation of CBD (94%), and controlled release properties. Niosomal cannabidiol retained the antineoplastic activity of the free agent, but noteworthy superior apoptogenic and inflammatory biomarker-modulating effects were established at equieffective exposure vs. the free drug. Specific alterations in key signaling molecules, implicated in programmed cell death, cancer cell biology, and inflammation, were recorded with the niosomal formulations.

## 1. Introduction

Niosomes have captured enormous scientific interest in the recent years, owing to their intrinsic favorable characteristics such as biocompatibility, non-immunogenicity, good tolerability, and low preparation cost, as well as to the technological advances they provide [1,2]. Niosomes are capable of increasing the stability of an encapsulated drug, to release it in a controlled way, to modify the pharmacokinetic profile, and to improve drug bioavailability [3,4]. Structurally denoted as vesicular systems, they comprise a central aqueous compartment, surrounded by a bilayer membrane, providing thereby encapsulation areas for both hydrophilic and hydrophobic compounds [5,6]. The non-ionic surfactants forming the bilayer membrane contribute to the improved chemical stability of dispersions (compared to liposomes), and to the simplified development process, with regard to constituents’ accessibility, handling, and storage requirements [7]. Another important asset of niosomes is the possibility to modify their structure to achieve targeted delivery [8,9], which is a highly advantageous approach in cancer treatment, determining less adverse reactions and superior therapy outcomes [10].

The subsequent vesicular modification also overcomes some of the limitations characteristic of vesicular carriers (including niosomes), such as short blood circulation time and rapid elimination by the mononuclear phagocyte system (MPS), which hamper their wider clinical application [11]. According to the relevant data on the topic [12,13,14,15], the aforementioned constraints are most often surmounted by the elaboration of “stealth” niosomes, using polyethylene glycol (PEG) to create a hydrophilic shell on the vesicular surface, serving as a protection shield and hindering their sequestration by the MPS [7].

The current study implements a different approach to achieve longer niosomal circulation time in the bloodstream using newly synthesized amphiphilic copolymers to impart steric stabilization. Star-shaped 3- and 4-arm star-shaped copolymers based on polyglycidol (PG) and poly(ε-caprolactone) (PCL) blocks and their linear analogue—ABA triblock copolymer (where A is PG and B is PCL blocks)—were synthesized by azide-alkyne *click* reactions with a subsequent removal of the protective ethoxyethyl groups. The hydrophobic segment (i.e., poly(ε-caprolactone)) would facilitate copolymers’ incorporation into the niosomal bilayer membrane, whereas the hydrophilic polyglycidol chains would provide the repulsive barrier function.

The developed plain (non-modified—N.M.) and copolymer-modified niosomes are loaded with cannabidiol (CBD), a non-psychotropic biologically active compound derived from *Cannabis sativa* and well-known for its antidepressant, neuroprotective, anti-seizure, anti-inflammatory, antioxidant, and antineoplastic properties [16,17,18]. Unfortunately, its diverse pharmacological effects cannot be fully exploited and translated to the clinic due to its unfavorable physicochemical and pharmacokinetic characteristics. The phytocannabinoid is a highly hydrophobic compound (aqueous solubility 0.1 μg/mL) [19], which is unstable under acidic environment (i.e., gastrointestinal tract) and characterized by a significant first-pass effect [20,21]. Altogether, these factors determine the observed low bioavailability (approx. 6%) after oral/oromucosal administration [22], which directs research efforts to explore other routes of administration or to develop nanoscale drug delivery systems for successful CBD delivery. Various nanocarriers such as nanoemulsion, polymer coated nanoparticles [23], transfersomes [24], nanostructured lipid carriers [25], polymeric micelles [26,27], liposomes [28,29], and ethosomes [30] have been already reported as effective CBD delivery platforms. However, liposomal carriers suffer from some disadvantages in terms of systemic delivery, such as low CBD encapsulation efficiency and short half-life; therefore, our paper provides an alternative technological approach in terms of both CBD entrapment and drug delivery carrier. Additionally, the niosomes as drug delivery systems per se are capable of overcoming some of the limitations associated with the above-mentioned nanocarriers, such as insufficient chemical stability (liposomes, transfersomes), and of maintaining superior physical stability compared to nanoemulsions due to their susceptibility to coalescence, or to NLC, in which lipid phase transitions can occur during the formulation or storage stage [31]. To the best of our knowledge, this is the first paper about cannabidiol encapsulation in niosomes (whether conventional or modified), which would undoubtedly enrich the research progress in this area.

The primary objective of this present investigation was to advance and thoroughly examine conventional and copolymer-modified niosomes as a promising platform for the systemic delivery of cannabidiol. The elaborated vesicles underwent comprehensive physicochemical and pharmacological evaluation, facilitating a comparative analysis of the influence of copolymer type and concentration on key physicochemical attributes, drug release kinetics, as well as cytotoxicity and pro-apoptotic and anti-inflammatory properties. Through these rigorous assessments, a deeper understanding of the potential benefits and implications of employing copolymer modifications in niosomal formulations for cannabidiol delivery was attained.

## 2. Materials and Methods

### 2.1. Materials

Glycidol (2,3-epoxypropanol, 96%, Sigma-Aldrich Schnelldorf, Germany), ethyl vinyl ether (99%, Sigma-Aldrich, Schnelldorf, Germany), p-toluenesulfonic acid (ACS reagent, ≥98.5%, Sigma-Aldrich, Schnelldorf, Germany), cyclohexanol (anhydrous ≥99.5%, Sigma-Aldrich, Schnelldorf, Germany), 4-pentynoic acid (95%, Acros Organics, Geel, Belgium), 4-dimethylaminopyridine (DMAP, ReagentPlus, ≥99%, Sigma-Aldrich, Schnelldorf, Germany), N-(3-dimethylaminopropyl)-N′-ethylcarbodiimide hydrochloride (EDC, commercial grade, powder, Sigma-Aldrich), triethylamine (TEA, ≥99.5%, Aldrich), methanesulfonyl chloride (≥99.7%, Aldrich), sodium azide (ReagentPlus, ≥99.5%, Sigma-Aldrich), sodium sulfate (≥99.99% trace metals basis, anhydrous, Sigma-Aldrich), AlCl_3_·6H_2_O (99%, Sigma-Aldrich), sodium hydrogen carbonate (anhydrous, ≥99.7%, Sigma-Aldrich), N,N,N′,N″,N″-pentamethyldiethylenetriamine (PMDETA, 99%, Sigma-Aldrich), copper (I) bromide (99.999% trace metals basis, Sigma-Aldrich), activated neutral Al_2_O_3_ (Fluka), methanol (ACS reagent, ≥99.8%, Sigma-Aldrich), diethyl ether (Merck, Darmstadt, Germany), heptane (s), ethyl acetate (Fisher Chemicals, Fisher Scientific GmbH, Schwerte, Germany ), and Tin (II) 2-ethylhexanoate (92.5–100.0%, Sigma-Aldrich) were used as received. Methylene chloride (>99.98%, Fisher Scientific, Schwerte, Germany) and ɛ- caprolactone (ɛ-CL; Sigma-Aldrich) were distilled over CaH_2_ before use. Propylene oxide (PO, 99%, Sigma-Aldrich) was dried over CaH_2_, distilled, and stored over a 4 Å molecular sieve before use. Toluene (>99.8%, Fisher Scientific, Schwerte, Germany) and tetrahydrofuran (THF, >99.5% Fisher Scientific) were dried by refluxing over a sodium-benzophenone mixture and subsequently distilled. Dimethylformamide (>99.99%, Fisher Scientific) was dried over diphosphorus pentoxide and distilled. Deionized water was obtained using a Millipore MilliQ system (Merck Millipore, Darmstadt, Germany) and additionally filtered through a 220 nm PTFE filter and a 20 nm cellulose filter. Poly(ε-caprolactone) diol, R[(CL)_9_-OH]_2_ (CAPA 2205, molecular weight of 2000, >99%, Cas. No 36890-68-3, Perstorp, Malmö, Sweden), poly(ε-caprolactone) triol, R[(CL)_6_-OH]_3_ (CAPA3201, molecular weight of 2000, >99%, Cas. No 37625-56-1, Perstorp), and poly(ε-caprolactone) tertaol, R[(CL)_2_-OH]_4_ (CAPA 4101, molecular weight of 1000, >99%, Cas. No 35484-93-6, Perstorp) were dried by azeotropic distillation of toluene before use. Ethoxyethyl glycidyl ether (EEGE) was synthesized as described elsewhere [32,33]. Synthetic details and characterization data for the polymer precursors are presented in the ESI (Schemes S1–S6, Figures S1–S15).

Cannabidiol (CBD) was a generous gift from PBG GLOBAL (https://pgb.global accessed on 21 September 2023. Span 20, Span 60, Span 80, Tween 60 and cholesterol were purchased by Sigma-Aldrich (FOT, Bulgaria). All solvents used in the experiments were of analytical grade.

### 2.2. Methods

#### 2.2.1. Synthesis of the Star-like 4-Armed Copolymer

Azide-terminated tetrafunctional PCL, R[(CL)_5_N_3_]_4_, (0.2570 g, 0.1275 mmol, 1 eq), and CuBr (0.736 g, 5.13 mmol, 40 eq) were added to a 50 mL round-bottom flask under an argon atmosphere. Dry THF (3 mL) was added via a syringe, and the solution was purged with argon and stirred vigorously for 20 min. Monoalkyne-terminated PEEGE and Cy-(EEGE)_23_-(PO)_7_-C≡CH (1.953 g, 0.514 mmol, 4 eq) were dissolved in dry THF (4 mL) and added to the PCL solution along with PMDETA (1.3300 g, 7.67 mmol, 60 eq). The *click* coupling reaction was carried out at 30 °C for 24 h. After completion of the reaction, the reaction mixture was cooled to RT, diluted with THF (30 mL), and filtered through a column filled with neutral alumina to remove copper complexes. The excess THF was evaporated; the crude product was dissolved in methanol (10 mL) and dialyzed against a methanol/water mixture (10:1 *v*/*v*, membrane, MWCO 8 kDa) for 72 h. The methanol was removed using a rotary vacuum evaporator, and the copolymer was recovered by freeze-drying. Yield: 0.667 g (37%); M_n_^HNMR^ = 17,200 g.mol^−1^, M_n_^SEC^ = 14,100 g.mol^−1^, M_w_/M_n_ = 1.17 (Figures S14 and S15). PEEGE blocks were derivatized into PG ones by treatment with AlCl_3_·6H_2_O as described elsewhere [34,35]. R[(CL)_5_(PO)_7_(EEGE)_23_]_4_ (0.60 g, 0.0348 mmol, 1 eq) was dissolved in methanol (4.36 mL, 156.97 mmol, 4500 eq) at 40 °C, and then AlCl_3_·6H_2_O (0.00842 g, 1 eq) was added under stirring. The hydrolysis was conducted at the same temperature for 48 h until complete disappearance of the methine proton signal at 4.75 ppm in the ^1^H-NMR spectrum (Scheme S15). The reaction mixture was filtered through Hyflo Super Cel diatomaceous earth, and then methanol was evaporated under reduced pressure.

Synthetic details and characterization data for the linear and star-like, 3-arm copolymers are presented in the Supplementary Materials (Schemes S1–S6, Figures S1–S15).

#### 2.2.2. Size Exclusion Chromatography (SEC)

Analyses were performed on Shimadzu Nexera HPLC chromatograph, equipped with a degasser, a pump, an auto-sampler, a RI detector, and three columns: 10 µm PL gel mixed-B, 5 µm PL gel 500 Å, and 50 Å. Tetrahydrofuran was used as the eluent at a flow rate of 1.0 mL.min^−1^ and temperature 40 °C. The sample concentration was 1 mg.mL^−1^, and SEC was calibrated with polystyrene standards.

#### 2.2.3. Proton Nuclear Magnetic Resonance (^1^H-NMR)

^1^H-NMR measurements were conducted on a Bruker Avance II spectrometer operating at 600 MHz using CDCl_3_ or DMSO at 25 °C.

#### 2.2.4. Preparation of Cannabidiol-Loaded Conventional and Sterically Stabilized Niosomes

Conventional and copolymer-modified cannabidiol-loaded niosomes were prepared by thin film hydration (TFH) method [36]. Chloroform solutions of niosomal constituents: nonionic surfactants (Span 20, Span 60, Span 80, an equimolar mixture of Span 60 and Tween 60) and cholesterol (30 and 40 mol % at total lipid 30 µmol/mL) and methanolic solution of the model drug—CBD (1.5 µmol/mL)—were subjected to rotary evaporation (Buchi, Germany) at 60 °C and 150 rpm. In the case of the sterically stabilized vesicles, the composition additionally comprised the investigated copolymers (0.5, 1, and 2.5 mol %), dissolved in chloroform/methanol (2:1 *v*/*v*). A subsequent step in the preparation process included the hydration of the formed film with phosphate-buffered saline (PBS) of pH 7.4 (5 mL) for 60 min at 60 °C under constant agitation (100 rpm). The resulting CBD-loaded niosomal dispersions were sonicated via probe sonicator (2 mm diameter) (Bandelin Sonoplus HD2200, Germany) at 30% amplitude and varying time intervals (2, 3, and 5 min) in a pulsatile manner (30 s on, 10 s off, 50 s on, 10 s off). The developed niosomes were kept at 4 ± 2 °C for subsequent investigation.

#### 2.2.5. Entrapment Efficiency

The experiment comprised an initial gel filtration of the elaborated niosomal dispersions via Sephadex G50 column (Pharmacia, Uppsala, Sweden) to separate the unentrapped CBD, followed by a liquid/liquid extraction. CBD-loaded niosomes were dissolved in an equivolume mixture of chloroform/methanol, to which aliquots of phosphate buffer (pH 7.4) were thereafter added. The formed dispersions were briefly vortexed, then subjected to centrifugation (5 min/6000 rpm). The concentration of CBD in the organic phase was determined spectrophotometrically (Shimadzu UV-1800) at λ = 274 nm, and the drug entrapment efficiency was calculated using the formula:

(1) $$EE\left(\%\right)=\frac{\mathrm{Amount}\;\mathrm{of}\;\mathrm{entrapped}\;CBD}{\mathrm{Total}\;\mathrm{amount}\;\mathrm{of}\;CBD}\times 100$$

#### 2.2.6. Size, Size Distribution, and Zeta Potential Evaluation

The size, size distribution, and zeta potential of elaborated blank and CBD-loaded niosomes (conventional and sterically stabilized) were assessed by Zetasizer NanoZS (Malvern Instruments, UK). The experiment was performed three-fold at 25 °C and scattering angle of 173°. The hydrodynamic diameters (D_h_) (±SD) represent the mean size of studied niosomes and were calculated applying the Stokes–Einstein equation:

(2) $$D_{h}=\frac{kT}{3\pi \eta D}\;\;$$

where k equals the Boltzmann constant, T is temperature (K), η is the solvent’s viscosity in accordance with temperature, and D represents the diffusion coefficient.

The zeta potential (ζ) of the vesicles was evaluated by electrophoretic light scattering measurements carried out on the above-mentioned instrument at a scattering angle of 173° at 25 °C. The ζ-potential was calculated using the Smoluchowski equation:

ζ = 4πηυ/ε(3)

where η is the solvent viscosity, υ is the electrophoretic mobility, and ε is the dielectric constant of the solvent.

#### 2.2.7. Cryogenic Transmission Electron Microscopy (cryo-TEM)

The morphology of the conventional and copolymer-modified CBD niosomes was visualized via cryogenic transmission electron microscopy using Tecnai F20 X TWIN microscope (FEI Company, Hillsboro, OR, USA) at 200 kV. For the preparation of cryo-samples, 3 µL of niosomal dispersions was placed onto copper grid (carefully removing the excess amount by filter paper) and instantly frozen in liquid ethane. After vitrifying, the specimens were placed in liquid nitrogen until examined at −178 °C. For the recording of the micrographs, a Gatan Rio 16 CMOS 4k camera (Gatan Inc., Pleasanton, CA, USA) was used, and the corresponding software (Gatan Inc., Pleasanton, CA, USA) provided the subsequent analysis.

#### 2.2.8. Carboxyfluorescein (CF) Release Studies

Carboxyfluorescein-loaded niosomes were prepared via TFH method, as described in Section 2.2.4. First, 80 mM of carboxyfluorescein-buffered solution (pH 7.4) was used as hydration medium, as at this concentration its fluorescence is self-quenched. Then, the non-encapsulated dye was separated by gel-filtration (Sephadex G50 column). Afterwards, 200 µL of carboxyfluorescein-loaded niosomes were added to 2 mL of PBS (pH 7.4). The solution fluorescence was evaluated after 24 h of incubation at 37 °C at λ_emm_ 520 nm and λ_ex_ 490 nm via Hitachi 7000 fluorescence spectrophotometer. To determine the total intensity, niosomes were lysed by addition of 100 µL of a 10% Triton X100 solution, and carboxyfluorescein leakage was calculated by applying the equation:

(4) $$CF\;leakage\left(\%\right)=\frac{\left(I_{t}\right)}{{(I}_{total})}\times 100$$

where I_t_ and I_total_ denoted the intensity at the determined time period and the total intensity, respectively.

#### 2.2.9. In Vitro Cannabidiol Release from Niosomes

Cannabidiol release from plain and optimal copolymer-modified niosomes was investigated by applying the dialysis method [37]. Two milliliters of the tested samples were placed in a presoaked dialysis membrane (MWCO 12,000–14,000, Sigma-Aldrich, Steinheim, Germany), and the sealed dialysis sac was immersed in a 100 mL acceptor medium—phosphate-buffered saline (PBS) of pH 7.4 and PBS of pH 7.4 + 20% albumin. To improve CBD solubility in PBS and ensure its detectability by UV–Vis, both of the release media contained 10% ethanol. The experiment was performed at 37 ± 0.5 °C and under continuous stirring (100 rpm), threefold. At stipulated time points, 2 mL samples of the release medium were taken and investigated for cannabidiol content spectrophotometrically at λ = 274 nm using a pre-built calibration curve with linearity in the range of 0.025 to 10 µg/mL (correlation coefficient R^2^ = 0.997).

The kinetics of cannabidiol release from the prepared niosomes was evaluated by fitting the data to zero order, first order, Higuchi model, and Korsmeyer–Peppas mathematical models. Linear regression analysis was used for zero and first order models, and non-linear fitting for Higuchi and Korsmeyer–Peppas models using DDSolver, a Microsoft Excel data analysis tool [38]. Based on the highest determination coefficient (R^2^), the best-fitting model was chosen.

#### 2.2.10. Physical Stability Studies

The physical stability of selected plain and copolymer-modified CBD-loaded niosomes was assessed by storage of the dispersions at 4 ± 2 °C for one month. The investigated parameters at the beginning and the end of the experiment included size, PDI, zeta potential, and CBD entrapment efficiency. The physical appearance of niosomal dispersions in regard to potential sedimentation or discoloration was also evaluated.

#### 2.2.11. Evaluation of Cytotoxicity of Cannabidiol and CBD-Loaded Conventional and Copolymer-Modified Niosomes

##### Cell Lines and Culture Conditions

The in vitro antiproliferative activity of the CBD-loaded niosomal formulations and free cannabidiol was assessed in a panel of tumor cell lines of different origin, namely urinary bladder carcinoma (T-24) and cutaneous T-cell lymphoma (HUT-78 and MJ). T-24 (ACC 376) cells were obtained from the German Collection of Microorgansims and Cell Cultures (https://www.dsmz.de/ (accessed on 15 June 2023)), and HUT-78 (TIB-161) and MJ (CRL-8294) cells were purchased by ATCC (https://www.atcc.org/ (accessed on 22 April 2022)). Cell cultures were cultivated in a growth medium RPMI 1640 supplemented with 10% fetal bovine serum (FBS) and 5% L-glutamine, and incubated under standard conditions of 37 °C and 5% humidified CO_2_ atmosphere. Urinary bladder cancer T-24 cells were kept in the logarithmic phase of growth by trypsinization two times a week. Suspension cultures of MJ and HUT-78 cells were refed 2–3 times a week by exchanging 30–50% of the cell suspension with fresh medium.

##### MTT Colorimetric Assay

The in vitro antiproliferative activity of the CBD niosomal formulations and free cannabidiol was evaluated using a validated methodology for assessing cell viability known as the Mosmann MTT dye reduction assay. Exponential-phased cells were harvested and seeded (100 μL/well) in 96-well plates at the appropriate density (3 × 10^5^ for the suspension cultures and 1.5 ×10^5^ for the adherent T-24 cells). Following a 24 h incubation, cells were exposed to five different doses of the nanocarriers with respect to cannabidiol concentration (135.7, 108.5, 81.4, 54.2, and 27.1 μM). After exposure time of 72 h, filter sterilized MTT substrate solution (5 mg/mL in PBS) was added to each well of the culture plate. A further 2–4 h incubation allowed for the formation of purple insoluble formazan crystals. The latter were dissolved in isopropyl alcohol solution containing 5% formic acid prior to absorbance measurement at 550 nm. Collected absorbance values were blanked against MTT and isopropanol solution and normalized to the mean value of untreated control (100% cell viability).

#### 2.2.12. Proteome Profiler Analysis of Apoptosis and Inflammation Related Signal Transduction Key Proteins

A series of immunoassay experiments were performed to monitor changes in the proteome profile of cells treated with the newly developed CBD formulations. Changes in the apoptotic and survival signaling, as well as in the inflammatory status of T-24 and MJ cells, in response to 24 h exposure to free cannabidiol and its niosomal compositions were tracked. Membrane-based sandwich immunoassays were conducted according to manufacturer’s instructions (Proteome Profiler Human Apoptosis Array Kit, R& D Systems; Proteome Profiler Human Cytokine Array Kit, USA, R&D Systems Ink). The arrays were visualized using a digital imaging system (Azure Biosystems C600, Dublin, CA 94568, USA), and densitometric analysis of the array spots was conducted using ImageJ^Ⓡ^ 1.8.0. software. The most prominent changes in the proteome were expressed graphically relative to untreated control and interpreted in a comparative manner relative to unformulated cannabidiol as a reference compound.

## 3. Results and Discussion

### 3.1. Synthesis of Copolymers

**Scheme 1:**
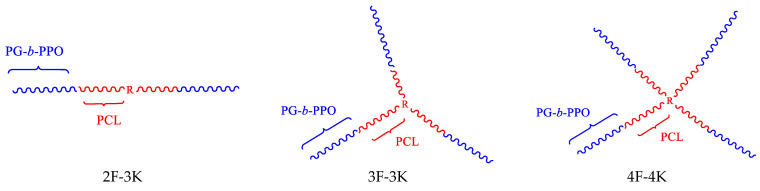
Chain architecture of the synthesized linear and star-like block copolymers. R represents a diol, triol, or tetraol residue. PPO is a short spacer of poly(propylene oxide).

A series of amphiphilic block copolymers comprising polyglycidol (PG) and poly(ε-caprolactone) (PCL) with different chain architecture—linear and star-like with 3 and 4 arms—were synthesized. The chain topology of the copolymers is schematically presented in Scheme 1.

**Scheme 2:**
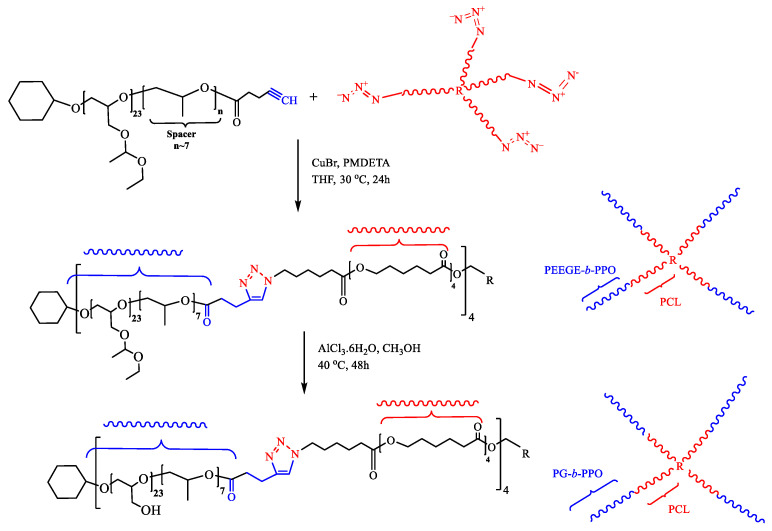
Schematic representation of the synthesis of R[(CL)_5_(PO)_7_(EEGE)_23_]_4_ star-like copolymer by copper-catalyzed azide-alkyne *click* reaction and subsequent deprotection leading to the amphiphilic R[(CL)_5_(PO)_7_(G)_23_]_4_ star-like copolymer. R represents a tetraol residue.

The copolymers were prepared via the recently introduced *click*-*chemistry*-based concept [33], enabling the synthesis of (co)polymers of precisely designed macromolecular characteristics and composition, as well as controlled length, topology, and well-defined positions of functional groups along the polymer chain. The synthetic approach is based on an azide-alkyne *click* reaction of monoalkyne functional poly(ethoxyethyl glycidyl ether) (PEEGE) and azide end-capped linear bi- or star-like tri- and tetrafunctional PCL and subsequent hydrolysis of the protective ethoxyethyl groups. The reaction steps for the 4-arm star-like copolymer are presented in Scheme 2.

**Figure 1:**
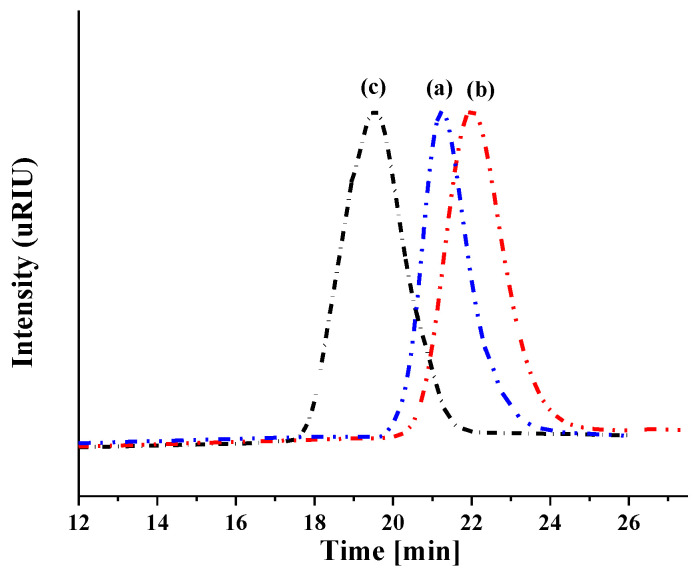
SEC traces of monoalkyne functional PEEGE (a), star-like tetraazidoPCL (b), and the 4- arm star-like copolymer R[(CL)_5_(PO)_7_(EEGE)_23_]_4_ (c). THF was used as the eluent at a flow rate of 1.0 mL.min^−1^ and temperature 40 °C. R represents a tetraol residue.

First, monofunctional PEEGE bearing a hydroxyl end group was prepared by ring-opening polymerization of EEGE. At the end of EEGE polymerization, a given amount of propylene oxide was added to extend the PEEGE block with a short poly(propylene oxide) (PPO) spacer. The introduction of the spacer has been shown to considerably increase the conversion and facilitate the esterification with pentynoic acid, thereby introducing alkyne functionality [33]. The other macroreagent—tetrazidoPCL—was obtained by a two-step procedure involving mesylation of the primary hydroxyl groups of PCL tetraol and azidation with NaN_3_. The *click* reaction was carried out in tetrahydrofuran under argon at 30 °C for 24 h using a CuBr/PMDETA catalytic complex. The resulting product was characterized by SEC and ^1^H NMR spectroscopy. The SEC trace of the product was shifted to lower retention time as compared to the traces of the two macroreagents (Figure 1), which indicated an effective coupling reaction. In the final step, the protective ethoxyethyl groups were cleaved at mild conditions, thereby converting the blocks of PEEGE into polyglycidol (PG).

The other two copolymers were synthesized following the same procedure using diazidoPCL and triazidoPCL to obtain linear and 3-arm star-like copolymers, respectively. Detailed description of the synthesis, functionalization, and characterization of all macroreagents, intermediates, and final copolymers is presented in the Supplementary Materials (Schemes S3 and S4, Figures S3–S6). Table 1 summarizes the composition, molar mass characteristics, and abbreviations of the intermediates and final copolymers. As seen from Table 1, the hydrophilic portion of the arms was of constant composition, whereas the hydrophobic one decreased from 9 to 5 monomer units. The copolymers differed in number of arms (2, 3, and 4), in topology (linear and star-like with 3 and 4 arms), as well as in total molar mass (from 5900 to 10,400).

For the aims of the present study, the star-like copolymers have a number of favorable properties and characteristics. Generally, the star-like copolymers are characterized by a more compact structure and smaller hydrodynamic sizes and radii of gyration than linear analogues with the same molar mass [39]. This implies that when intercalated in the bilayer membrane, they would introduce less disturbance and would not severely alter the bilayer structure and properties. Furthermore, the amphiphilic star-like copolymers have higher critical micelle concentrations, so their self-assembly is hindered compared to their linear analogues [39]. The beneficial effect would be that in the niosomal preparations, formation of self-assembled copolymer structures (e.g., micelles) would be hampered. Last but not least, at equal molar contents, the star-like copolymers would provide more hydrophilic chains grafted on the bilayer membrane, which would aid the formation of a denser and thicker layers around the membranes, bringing about increasing stability and prolonging blood circulation time.

### 3.2. Characterization of Conventional and Copolymer-Modified Niosomes

Conventional and copolymer-modified niosomes were prepared via TFH method, investigating the impact of composition variables (type of surfactants, cholesterol concentration, and type and concentration of amphiphile copolymers) and process parameters (sonication time and cycles) on their main physicochemical characteristics, aiming to derive a formulation characterized by high CBD entrapment efficiency and size distribution patterns suitable for systemic delivery. The obtained results are presented in Table 2.

Particle size is one of the important parameters to be considered during the development of nanocarriers intended for systemic delivery. Generally, sizes between 100 and 300 nm are suitable for this route of administration, as these dimensions would facilitate nanocarriers’ deposition in tumors due to the enhanced permeability and retention (EPR) effect [40]. Our preliminary studies involved evaluation of the effect of sonication time (2, 3, 5 min) and number of cycles (30 s or 50 s sonication with 10 s pause) on niosomal dimensions. The below-mentioned discussed results are obtained by sonicating the formulations for 2 min in a pulsatile manner (30 s sonication, 10 s pause). As can be seen from the presented results, the selected preparative parameters lead to the production of small vesicles with sizes in the 130–250 nm range and a polydispersity index below 0.4, suitable for systemic administration (Table 2, Figures S16 and S17 in the Supplementary Materials). The increasing of sonication time and/or number of cycles led to the formation of vesicles outside of the desired size range (less than 100 nm). For reference purposes, unsonicated formulations based on the optimal composition (Span 60:Tween 60:Chol, 3.5:3.5:3) (blank and CBD-loaded, formulations S7 and S8) were also prepared and were used as a model to evaluate the impact of type and concentration of the investigated copolymers on the main physicochemical characteristics without the effect of subsequent energy input in the systems.

Among the screened Span surfactants, Span 20-based niosomes (S1) exhibited greater size, whereas Span 80-ones (S2) exhibited the smallest. These outcomes may be related to the lower hydrophobicity of Span 20 (HLB value of 8.6 vs. 4.7 of Span 60 and 4.3 of Span 80), which leads to the formation of vesicles with larger size [41]. The addition of Tween 60 in the composition (S4) decreases vesicles’ dimensions as compares to S3, which may be explained by the occurred highly packed state of molecules within the vesicles [42].

The type of surfactants also affects entrapment efficacy, as evident from the presented data (Table 2). Among the screened surfactants at constant cholesterol concentration (S1–S4), the highest CBD encapsulation (85.2%) was achieved using equimolar ratio of Span 60 and Tween 60 (S4). This may be related to the high phase transition temperature and long chain (C-18) structure of Span 60, which determines the observed higher EE values (80.3% for S3 and 85.2% for S4). Additionally, the inclusion of Tween 60 in S4 further enhances CBD loading by solubilizing the phytochemical, probably due to the occurrence of hydrogen bonds with its phenolic groups with OH groups of Tween 60.

The effect of cholesterol concentration on vesicles’ characteristics was also assessed (S4 and S5). Higher cholesterol content (40 mol %, formulation S4) determined lower CBD encapsulation (85.2% vs. 93.2% for S5), probably arising as a consequence of the competing mechanisms between both hydrophobic molecules. Larger vesicles were also formed at this concentration (vs. 30 mol % cholesterol—S5), which may be attributed to the rearrangement of the niosomal membrane structure. Increasing cholesterol content and respectively decreasing the non-ionic surfactants ratio determines the increased hydrophobicity of the niosomal membrane. The latter leads to deformation of the acyl chains, high surface tension, enhanced bilayer fluidity, and ultimately an increase in niosomal size as an adjusting mechanism to impart vesicular stability following the occurred structural alterations [43,44].

As evident from the presented data, inclusion of cannabidiol and copolymers also affects niosomal size. All copolymer-modified blank vesicles, as well as the unsonicated conventional ones, are characterized by larger sizes compared to their corresponding CBD-loaded counterparts (Table 2). One possible explanation may be the formation of a more condensed structure, resulting from the accommodation of CBD in the niosomal bilayer and its interaction with hydrophobic surfactants segments. In the case of the conventional sonicated niosomes (S5 and S6), the dependence was not observed, however, with a size difference between blank and drug-loaded niosomes of approx. 10%.

The copolymer-containing niosomes (both blank and CBD-loaded) are characterized by larger size than the corresponding conventional niosomes (cf. formulations S6 with formulations S9–S11, S13–S15, and S17–S19 and S5 with S12, S16, and S20 in Table 2 and Figure S17 in the Supplementary Materials). Apparently, the incorporation of the rigid, crystalizable PCL moieties induce formation of less curved membranes and, hence, larger-in-size vesicles. In addition, the protective layer around niosomes that is formed by the hydrophilic component of the copolymers—polyglycidol chains—may also contribute to the overall size increase. The trend for increasing of the niosome size with copolymer content from about 220 to 250 nm, with somewhat deviating behavior of formulations S17 and S18, can be explained by the increasing density and thickness of the protective polyglycidol layer. ζ potenial is not affected upon incorporation of copolymers. With respect to the EE% values, a slight decrease in the CBD entrapment upon the addition of copolymers and sonication was observed, but with corresponding values still around and above 90%.

**Figure 2:**
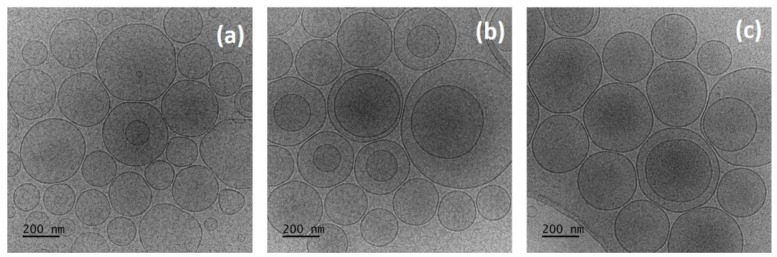
Cryo-TEM images of non-modified (N.M.) (**a**) and 2.5 mol % polymer-modified Tw60:Sp60:Chol (3.5:3.5:3) 3F-3K (S16) (**b**) and 4F-3K (S20) (**c**).

The impact of the investigated copolymers on niosomal morphology was also assessed, investigating for this purpose the formulations at their highest content (2.5 mol %) compared to nonmodified ones. Representative micrographs are shown in Figure 2. The obtained cryo-TEM images reveal the formation of well-separated spherical vesicles with intact membranes of 5–8 nm thickness and size distribution (based on observation of at least 100 particles per sample) in line with the conducted DLS analysis (see also Figure S18 in the Supplementary Materials). As observed from a large collection of micrographs, the addition of copolymers in concentration up to 2.5 mol % does not compromise niosomal vesicular structure. The images are dominated by mostly unilamellar vesicles. Small fractions of bilamellar niosomes were occasionally observed. It should be emphasized that openings in the bilayer membrane, perforations, bilayer fragments, small micelles, or other more complex morphologies were not found, which is an indication of a preserved vesicular integrity. The latter is an essential requirement to achieve controlled drug release profile and longer blood circulation time, which the study aimed to do.

### 3.3. Carboxyfluorescein Release from Conventional and Copolymer-Modified Niosomes

**Figure 3:**
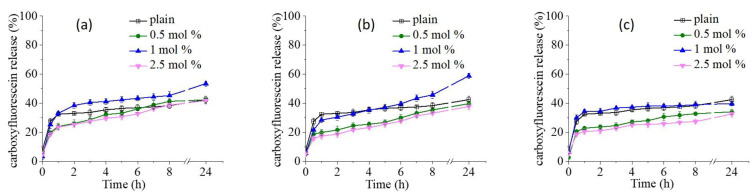
Carboxifluorescein release from modified by 2F-3K (**a**), 3F-3K (**b**), and 4F-3K (**c**) niosomes at various copolymer contents. The release from unmodified niosomes (plain) is given by open squares in (**a**–**c**).

Carboxyfluorescein release studies are among the most exploited approaches to evaluate vesicular nanocarriers’ permeability. The molecule of CF is hydrophilic, and it is believed to be released from the internal water pools mainly through transient pores and defects that are spontaneously formed in the bilayer [45,46,47]. In the current experiment, CF release from niosomes was assessed at a physiologically relevant temperature (37 °C), outlining in a comparative aspect the influence of copolymers’ incorporation, type, and concentration on this process. The results are presented in Figure 3 as CF release in % versus time for each copolymer at various contents—0.5, 1.0, and 2.5 mol %. The leakage profile of unmodified niosomes is given in each figure. The results show that the CF release is not strongly influenced by the incorporation of the copolymers. However, there are detectable differences in the membrane permeability, which are related with the content rather than with the copolymer type and chain architecture. As observed from the presented graphics, CF release from modified niosomes was not directly proportional to the content of the incorporated copolymers: the highest dye release was invariably estimated at 1 mol % of copolymer content, whereas lower (0.5 mol %) and higher (2.5 mol %) contents exerted a stabilizing effect on membrane’s permeability. Assuming a mechanism of leakage through spontaneously formed transient pores or defects in the membrane, the slightly enhanced or reduced leakage from the copolymer-modified niosomes can be interpreted in terms of stabilizing the pores/defects or reducing the probability of pore formation, respectively. As all investigated copolymers exhibited a stabilizing effect on the membrane’s permeability at 2.5 mol %, the assessed physicochemical characteristics (lowest size, highest CBD encapsulation) gave us reason to proceed with the experiments with the 3F-3K-modified niosomes.

### 3.4. In Vitro Release of Cannabidiol from Optimal Plain and Copolymer-Modified Niosomes

**Figure 4:**
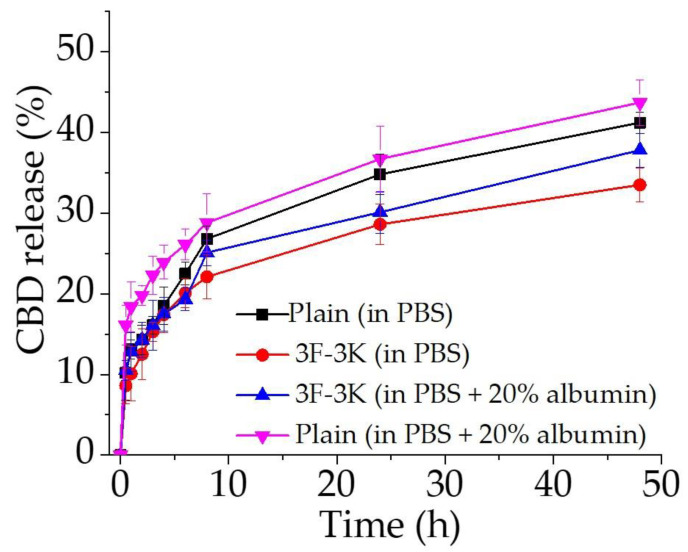
CBD cumulative release from plain or 3F-3K (2.5 mol %) (S16)-modified optimal niosomal formulations as a function of time at 37 °C in PBS (pH 7.4) or PBS + 20% albumin.

In line with the carboxyfluorescein leakage experiments are the results from the conducted in vitro release studies of CBD. Unlike CF, CBD is highly hydrophobic and, most probably, solubilized in the niosomal membrane as the most suitable encapsulation site for hydrophobic substances provided by the niosomes. Evident from the results presented in Figure 4 is that niosomes, modified by the 3F-3K copolymer, are able to release their cargo in a sustained manner for a prolonged time. Besides the beneficial effect of inclusion of Tween 60 resulting in enhancement of CBD loading, probably due to occurrence of hydrogen bonding (see above), the strong interactions with the hydrophobic surfactant segments and PCL moieties of the copolymer may also bring about a slowing down of the release of CBD. In order to obtain an idea of the behavior of the modified niosomes in physiological conditions, we followed the release of CBD after adding of 20% albumin in the acceptor phase. As a result, a slight increase in released CBD is observed, which may arise from the formed hollows upon albumin absorption onto the vesicular bilayer and the enhanced porosity induced thereby. However, the release of CBD from the modified niosomes was lower than that of the unmodified vesicles in phosphate buffer, and especially compared to the release profile of unmodified vesicles in the presence of 20% albumin, effectively proving the stabilizing role of the polymer shell.

The data from the in vitro release profiles was fitted to various release kinetics models to evaluate the release mechanism of cannabidiol from plain and copolymer-modified niosomes. The results showed that all the formulations were best explained by Korsmeyer–Peppas model (plots demonstrated the highest coefficient of determination), with diffusion exponent values bellow 0.45 indicating Fickian diffusion mechanism, where the release of cannabidiol is controlled by diffusion through the intact niosomal membrane [48]. (Table 3). These findings explain the slower drug release from the modified niosomes, where the presence of the polymer layer around the niosomal membrane increases the distance for diffusion. In addition, the results obtained from the in vitro release indirectly provide an idea about the stability of the systems in physiologically relevant conditions, since there is no difference in the release profiles and kinetics obtained in PBS medium + 20% albumin compared to solely PBS medium, which indicates the good steric stabilization provided by the 3F-3K copolymer incorporated in the membranes, which is a prerequisite for prolonged circulation of the modified niosomes upon systemic administration.

### 3.5. Physical Stability Evaluation

Since niosomes, like other vesicular systems such as liposomes, are inherently unstable, we monitored their storage stability in terms of size, size distribution, and amount of encapsulated CBD over a period of 1 month at 4 °C. As evident from the obtained data (Table 4), both plain and 3F-3K-modified niosomes are stable under the tested storage conditions, as there is no evidence of changes in the investigated formulation’s parameters. This observation can explain the stabilizing effect of cholesterol and the incorporated copolymer preventing thermal contraction or fusion of the vesicles [49].

### 3.6. Cytotoxicity Studies

#### 3.6.1. MTT—Assay

The cytotoxicity of the newly developed CBD-loaded niosomes was assessed in a series of MTT experiments against three human tumor models, namely T-cell lymphoma (MJ), Sézary syndrome (HUT-78), and urothelial carcinoma (T-24), in a comparative manner with unformulated cannabidiol (applied as ethanol solution) (Table 5). The tumor models were chosen based on their clinical relevance. Cutaneous T-cell lymphoma is a very rare malignant disease in humans with a frequency of less than 1 per 100,000 people, thus being assigned as an orphan disease. Primary cutaneous T-cell lymphomas (CTCLs) vary in form from those of a very indolent type to extremely aggressive malignancies with bad prognosis. CTCLs are rare diseases with an annual incidence of around seven cases per million population [50]. The most common CTCLs are mycosis fungoides (MF) and Sézary syndrome (SS), which account for around 50% of all primary CTCLs. In 2018, bladder cancer (urothelial carcinoma) was ranked twelfth with regard to worldwide diagnosis of malignancies. At the time point of diagnosis of bladder cancer, approximately 75% of patients present with a non-muscle-invasive disease, while the remaining 25% show invasion of tumor cells in the muscle layer of the bladder wall. Many patients with non-muscle-invasive tumors suffer from reoccurrence after successful transurethral resection and need additional local treatments [51]. According to our findings, least responsive to a 72 h treatment were the cutaneous T-cell lymphoma tumor models MJ, followed by the Sézary-syndrome-derived HUT-78, whereby the copolymer-modified niosomes (3F-3K, 4F-3K) yielded similar half-inhibitory concentrations, exceeding 1.5 to 3 times that of the free drug. The estimated IC_50_ values for the plain formulation were somewhat higher in all of the screened cell lines. These general trends in CBD potency based on its formulation appeared to also be valid in the malignant epithelial (T-24 urothelial bladder carcinoma), which showed higher overall chemosensitivity.

Nevertheless, the cytotoxic potential of CBD regardless of its formulation is a function of its amount present in the cellular milieu. Therefore, the kinetics of its time release should in any case be taken into consideration, being the major determinant of the drug availability in the culture medium. According to the cumulative release profiles of the three niosomal preparations (Figure 4), no more than 50% of their cargo would be released by 72 h of treatment, which may be liable for their inferiority relative to the free drug performance that we observed in the conducted MTT studies.

#### 3.6.2. Proteome Profiler Analysis after Treatment with Free or Niosomal CBD

**Figure 5:**
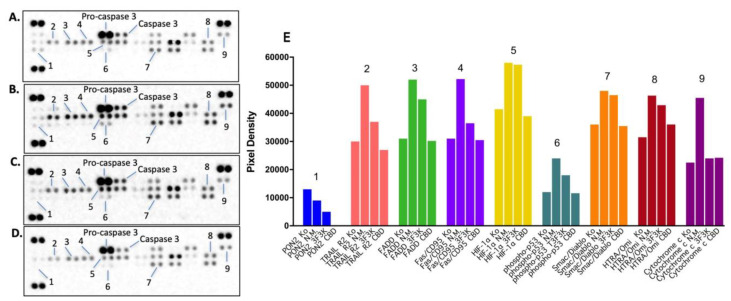
Changes in expression levels of apoptosis-related proteins in T-24 cells following treatment with CBD-loaded plain (N.M) (**B**) and 3F-3K-modified niosomes (**C**) and free CBD (**D**), as compared to untreated control (**A**). Cells were exposed to equieffective concentrations (IC_50_) of free cannabidiol and its niosome formulations for 24 h, following which a human proteome profiler assay was performed according to manufacturer’s instructions. Further densitometric analysis of the array spots was conducted using ImageJ software, and the most prominent changes in the proteome were expressed graphically (**E**). Legend: 1—PON2; 2—TRAIL R2; 3—FADD; 4—Fas/CD95; 5—HIF-1α; 6—phospho-p53; 7—Smac/Diablo; 8—HTRA/Omi; 9—Cytochrome C.

**Figure 6:**
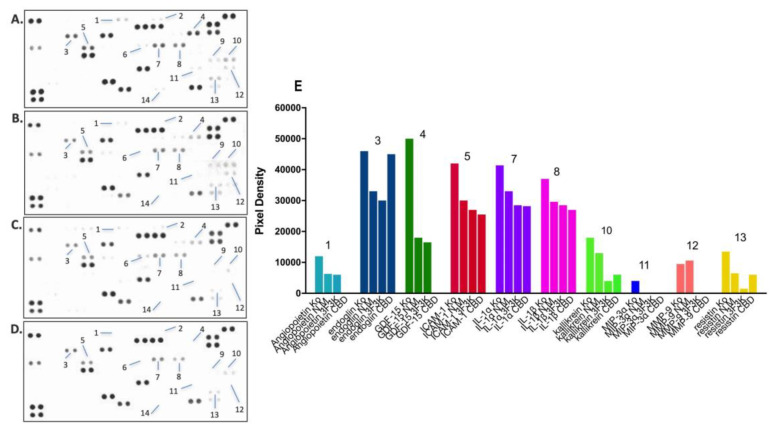
Changes in expression levels of inflammation-related proteins in T-24 cells following treatment with N.M (**B**), 3F-3K (**C**), CBD (**D**), as compared to untreated control (**A**). Cells were exposed to equieffective concentrations (IC_50_) of free cannabidiol and its niosome formulations for 24 h, following which a human cytokine profiler assay was performed according to manufacturer instructions. Further densitometric analysis of the array spots was conducted using ImageJ software, and the most prominent changes in the proteome were expressed graphically (**E**). Legend: 1—Angiopoietin-1; 2—BDNF; 3—endoglin; 4—GDF-15; 5—ICAM-1; 6—IGFBP-3; 7—IL-1α; 8—IL-1β; 9—I-TAC; 10—kallikrein 3; 11—MIP-3α; 12—MMP-9; 13—resistin; 14—Thrombospondin-1.

**Figure 7:**
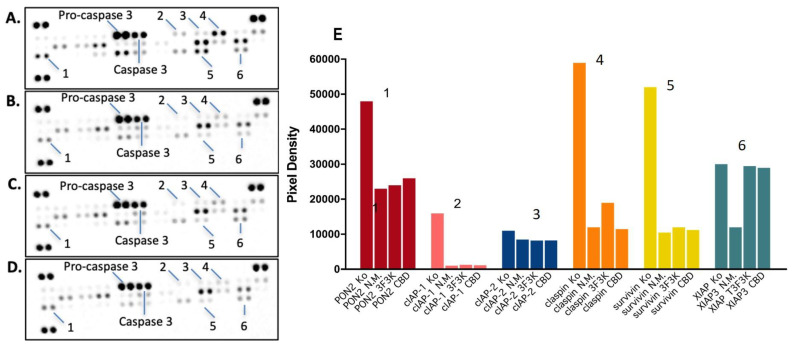
Changes in expression levels of apoptosis-related proteins in MJ cells following treatment with N.M (**B**), 3F-3K (**C**), and CBD (**D**), as compared to untreated control (**A**). Cells were exposed to equieffective concentrations (IC_50_) of free cannabidiol and its niosome formulations for 24 h, following which a human proteome profiler assay was performed according to manufacturer’s instructions. Further densitometric analysis of the array spots was conducted using ImageJ software and the most prominent changes in the proteome were expressed graphically (**E**). Legend: 1—PON2; 2—cIAP-1; 3—cIAP-2; 4—claspin; 5—survivin; 6—XIAP.

**Figure 8:**
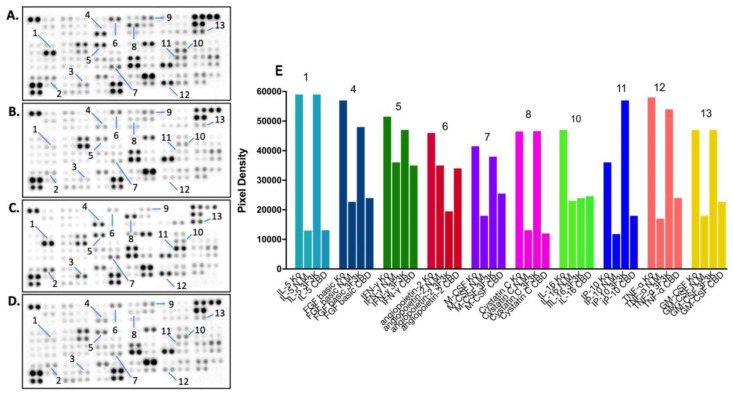
Changes in expression levels of inflammation-related proteins in MJ cells following treatment with N.M (**B**), 3F-3K (**C**), CBD (**D**), as compared to untreated control (**A**). Cells were exposed to equieffective concentrations (IC_50_) of free cannabidiol and its niosome formulations for 24 h, following which a human cytokine profiler assay was performed according to manufacturer instructions. Further densitometric analysis of the array spots was conducted using ImageJ software, and the most prominent changes in the proteome were expressed graphically (**E**). Legend: 1—IL-5; 2—Osteopontin; 3—Tarc; 4—FGF basic; 5—IFN-γ; 6—angiopoietin-2; 7—M-CSF; 8—Cystatin C; 9—BDNF; 10—IL-16; 11—IP-10; 12—TNF-α; 13—GM-CSF.

Albeit devoid of psychoactive effects, free cannabidiol has been demonstrated to exert pronounced anti-inflammatory, immunomodulating, neuroprotective, and proapoptotic activities through various mechanisms. Using proteome profiling, we sought to examine potential changes in the pharmacodynamic profile of the free CBD upon its niosomal formulation, applied as a beneficial strategy to optimize its unfavorable physicochemical and biopharmaceutical properties. We were able to simultaneously monitor changes in the patterns of protein expression of multiple key signaling mediators related to inflammation and apoptosis in bladder carcinoma (T-24) and cutaneous T cell lymphoma (MJ) cell lines, exposed to equieffective concentrations of either free or niosome-encapsulated cannabidiol (Figure 5, Figure 6, Figure 7 and Figure 8).

For the mechanistic studies of our new encapsulated cannabidiol formulations, we selected two in vitro tumor models: urothelial-cancer-derived T-24 cells and mycosis-fungoides-derived MJ cells in order to get specific data about changes in apoptotic-related and inflammation-related signaling proteins. The urinary bladder cell line T-24 represents one common solid tumor form, namely non-muscle invasive bladder cancer. This human solid tumor disease is less abundant with local inflammatory reactions, and even its treatment is directed to the use of immunostimulating agents such as BCG applied locally. Intravesical immunotherapy with Bacillus Calmette-Guérin (BCG) has been the standard of care for patients with high-risk non-muscle invasive bladder cancer (NMIBC) for over four decades. Despite its success as a cancer immunotherapy, disease recurrence and progression remain common. Current efforts are focused on developing effective and well-tolerated alternatives to BCG and salvage bladder preservation therapies after BCG has failed. One of these alternatives could be cannabidiol and especially its encapsulated niosomal formulations [52]. Mycosis fungoides is just the opposite example, because it is manifested by local inflammatory skin lesions, which can be explained even by the Th2 origin of the malignant cells [53].

The apoptotic response of both malignant cell types revealed several common as well as cell-line-specific changes in the expression levels of signaling and regulatory proteins in programmed cell death (Figure 5 and Figure 7). Neither of the proteomic arrays showed activation of the executory pro-caspase 3; however, the likelihood of this event is anticipated to be low in experimental settings with a short 24 h exposure time, given the distal occurrence of chromatin condensation and nuclear fragmentation in the apoptotic pathway. Both cell lines responded with consistent down-regulation of the paraoxonase PON2 (a membrane-bound enzyme that protects cells from oxidative stress), which was most pronounced in the CBD-treated T-24 carcinoma cells (leading to complete disappearance of the spot signal) (Figure 5), and the MJ sample exposed to the non-modified niosomal formulation N.M. (Figure 7).

Interestingly, several proapoptotic proteins maintained a unique pattern of induction among the T-24 treatment groups (Figure 5), whereby cannabidiol performance improved upon niosomal encapsulation in the order: CBD < 3F-3K < N.M. The most potent formulation, N.M., prompted a nearly two-fold increase in the expression levels of the “death” receptors TRAIL-2 and Fas/CD95, which are members of the TNFR superfamily and trigger the extrinsic apoptotic pathway. It was also able to induce their downstream adaptor protein FADD. Most importantly, the same CBD composition doubled the cytosolic release of cytochrome C, which is a major recruiter and activator of procaspase-9 in the alternative intrinsic mitochondrial pathway of apoptosis. Superior to free CBD was the augmenting effect of both its carriers (N.M. and 3F-3K-modified) on the cellular levels of Smac/diablo, another mitochondrial protein functioning as a key promotor of caspase-9 activation. Both niosomal formulations effectively increased the active phosphorylated form of the master p53 tumor suppressor, whose levels, on the other hand, remained unaltered in the free CBD treatment sample.

In the MJ lymphoma cells, the proteome profiles in all treatment groups were rather similar, revealing an isolated reduction in a cluster of related proteins, all acting as potent inhibitors of apoptosis: cIAP-1, cIAP-2, XIAP, and survivin (Figure 7). Within the bladder carcinoma cell line, a slightly better efficacy in depleting these survival factors was shown by the plain N.M. CBD composition (3- and 2-fold reduction in survivin and XIAP levels, respectively). Quantitative analysis also revealed a drastic decrease in the checkpoint protein Claspin, required for the DNA replication and cell cycle progression.

The inflammatory response to treatment in the cutaneous T-cell lymphoma model indicated a similar trend in CBD potency, depending on its formulation (Figure 8). Thereby, N.M. exerted the most potent down-regulating effect on the cytokinome (i.e., the set of cytokines), strongly implicated in inflammation (IL-5, IFN-γ and its inducible protein IP-10, Cystatin C, IL-16, and most importantly, TNF-α). Furthermore, a notable decline was registered in the expression levels of both the constitutive macrophage-specific growth factor M-CSF and the inducible-upon-inflammation GM-CSF cytokine that mediates a wider spectrum of stimulating activity on the proliferation and maturation of both monocyte and myeloid lineages. A high degree of reduction in the fibroblast growth factor FGF was induced by both N.M.-formulated and free cannabidiol, whereas 3F-3K-modified niosomes had the most pronounced inhibitory effect on angiopoietin-2, a multifaceted cytokine playing a central role in angiogenesis and inflammation. The reduction of the expression levels of cytokines and especially IL-5 may beneficially influence the skin lesions seen in patients with mycosis fungoides as mentioned in recent concepts of the molecular pathology and treatment options for CTCL [50].

CBD-induced changes in the expression profiles of the T-24 cyto- and chemokinome were less distinctive of its formulations, possibly due to the adherent nature of the cells, the complexity of their cross-talk interactions, and the hindrance in the long-distance cellular communication. Both free and niosomal cannabidiol reduced the expression of the major neutrophil- (IL-1α) and macrophage-recruiting (IL-1β) proinflammatory cytokines in a likewise manner, as well as the levels of the adherent molecule ICAM-1 (Figure 6). The 24 h exposure to the free drug and its 3F-3K-modified niosomal formulation resulted in complete disappearance of the spot signals for the macrophage inflammatory protein (MIP)-3α and the MMP-9 collagenase. The matrix metalloproteinases (MMPs) are known to contribute to the invasive and metastatic growth of malignant tumors [54]. Hence, the complete suppression of these proteins by elaborated 3F-3K niosomal formulation will probably reduce the invasive and metastatic potential of the urothelial cancer cells. The most prominent reduction in the expression levels of endoglin (proangiogenic factor of key importance to tumor growth, survival, and metastasis), kalikrein (a component of the kininogenic system in inflammation and blood pressure regulation), and resistin (an adipose-derived proinflammatory hormone) was also attained under the action of the 3F-3K-niosome-entrapped cannabidiol. Both CBD formulations produced a similar silencing effect on the angiopoietin and the GDF-15 stress-responsive growth/differentiating factor levels (a ca. 2- to 3-fold decrease), while free CBD yielded a complete depletion of both angiogenic and growth factors.

The presented comparative analysis of the proteomic imprints of both malignant cell lines revealed a stronger modulating activity of the niosome formulation on CBD apoptosis-inducing properties, whereby both delivery systems (especially the non-modified variant) induced the extrinsic and intrinsic apoptotic pathways in T-24 cells to a greater extend as compared to the free drug. In the MJ-treated cells, the same N.M. formulation was proven to be equal or superior to cannabidiol in regard to its anti-inflammatory potential, whereas treatment efficacy among the groups varied in the T-24 tumor model. Moreover, the accurate evaluation of CBD activity in its niosomal forms also requires taking into account its release kinetics (Figure 4), suggesting an incomplete availability of the loaded substance in cell media at the time of treatment. Upon the 24th hour of niosome formulation and cell treatment, both the plain N.M. niosomes and the 3F-3K-modified compositions would have released no more than 25–35% of their CBD cargo, rendering the treatment concentration in the conducted immunoanalytic studies approximately one third of the estimated IC_50_ value. Therefore, the actual net boosting effect of niosome formulation on CBD biological activity appears to be much greater after all.

## 4. Conclusions

In this work, niosomes modified with newly synthesized amphiphilic linear or star-shaped polyglycidol-poly(ε-caprolactone)-based copolymers were developed as a delivery platform for the systemic administration of cannabidiol. The obtained results revealed the feasibility of all investigated copolymers to provide steric stabilization of the vesicles in a physiologically relevant environment. The formulation based on Tween 60:Span60:Chol (3.5:3.5:3 molar ratio) modified with 2.5 mol % star-shaped 3-arm copolymer was characterized by high CBD entrapment efficiency (94%), size suitable for parenteral administration (235 nm), and controlled drug release; therefore, it was subjected to further pharmacological evaluation. Summarizing our findings related to cytotoxicity and the changes in the expression levels of apoptosis-related and inflammation-related proteins indicate that encapsulated CBD formulations may cause significantly higher increase of death receptor signaling elements such as TRAILR2, FADD, and Fas/CD95 in urothelial cancer cells T-24. Interestingly, the same is true for HIF-1α. The expression level of PON2 was substantially lower in T-24 cells, thus indicating that they are not adapted to high free radical stress (e.g., in case of local inflammation). CBD-containing formulations lead to significant reduction of adhesion molecules such as ICAM-1. CBD in the form of solution or encapsulated in 3F-3K-modified niosomes can completely inhibit the expression of MMP-9, thus most probably reducing the invasive and metastatic potential of the urothelial cancer cells.

Our data about the mycosis fungoides CTCL-derived MJ cells subjected to treatment with CBD and its niosomal formulations provides evidence that CBD, irrespective of the formulation, can reduce the expression level of PON2, thus seriously limiting the tolerance to free radical presence in the inflammatory microenvironment. Similar changes were seen in the levels of the cellular inhibitor of apoptosis (cIAP-1) and survivin, which can increase the chemosensitivity of the malignant CTCL cells. These findings suggest that the elaborated amphiphilic copolymer-modified niosomal formulations are promising platforms for cannabidiol delivery as they preserve its low micromolar cytotoxicity in cancer cells and augment its pro-apoptotic and anti-inflammatory effects, and warranting further investigation to elucidate the precise molecular mechanisms driving these effects.

## Figures and Tables

**Table 1:** Composition and molecular characteristics of amphiphilic poly(ε-caprolactone) (PCL) and polyglycidol (PG)-based copolymers with linear and star-shaped architectures.

Copolymer			M_n_ ^a^ (g/mol)	M_w_ ^b^ (g/mol)	M_w_/M_n_ ^b^
Topology	Composition ^c,d,e^	Abbreviation			
Linear	R[(CL)_9_(PO)_7_(EEGE)_23_]_2_		9600	13,200	1.20
Linear	R[(CL)_9_(PO)_7_(G)_23_]_2_	2F-3K	5900	-	-
Star-like, 3-armed	R[(CL)_6_(PO)_7_(EEGE)_23_]_3_		13,400	17,700	1.22
Star-like, 3-armed	R[(CL)_6_(PO)_7_(G)_23_]_3_	3F-3K	8300	-	-
Star-like, 4-armed	R[(CL)_5_(PO)_7_(EEGE)_23_]_4_		17,200	14,100	1.17
Star-like, 4-armed	R[(CL)_5_(PO)_7_(G)_23_]_4_	4F-3K	10,400	-	-

^a^—number-averaged molar mass from ^1^H NMR. ^b^—weight-averaged molar mass from SEC; M_w_/M_n_—molar mass distribution from SEC; ^c^—determined from ^1^H NMR; ^d^—CL, PO, EEGE, and G denotes monomer units of poly(ε-caprolactone), poly(propylene oxide), poly(ethoxyethyl glycidyl ether), and polyglycidol, respectively; ^e^—R represents a diol, triol, or tetraol residue.

**Table 2:** Composition, physicochemical characteristics, and entrapment efficacy of CBD-loaded niosomes (conventional and modified).

Sample	SF:Chol (mol:mol)	Copolymer (mol %)	D_h_ (nm) ± SD	PDI ± SD	ζ-Potential (mV) ± SD	EE (%) CBD
Conventional niosomes						
S1	Sp20:Chol (6:4)	-	230 ± 4.2	0.26 ± 0.05	−13.3 ± 1.9	65.8 ± 1.7
S2	Sp80:Chol (6:4)	-	180 ± 3.2	0.22 ± 0.05	−26.6 ± 2.2	64.7 ± 1.6
S3	Sp60:Chol (6:4)	-	186 ± 2.8	0.4 ± 0.02	−12.9 ± 1.7	80.3 ± 0.7
S4	Tw60:Sp60:Chol (3:3:4)	-	177 ±2.3	0.39 ± 0.03	−11.9 ± 0.5	85.2 ± 2.3
S5	Tw60:Sp60:Chol (3.5:3.5:3)	-	150 ± 1.1	0.35 ± 0.07	−10.3 ± 1.6	93.2 ± 2.1
S6	Tw60:Sp60:Chol (3.5:3.5:3) (blank)	-	133 ± 1.7	0.31 ± 0.09	−11.2 ± 1.5	-
S7	Tw60:Sp60:Chol3.5:3.5:3(unsonicated)	-	387 ± 5.3	0.32 ± 0.02	−11.9 ± 2.1	93.5 ± 1.8
S8	Tw60:Sp60:Chol 3.5:3.5:3 (blank) (unsonicated)	-	489 ± 3.8	0.34 ± 0.06	−12.3 ± 2.3	-
Niosomes modified with 2F-3K						
S9	Tw60:Sp60:Chol 3.5:3.5:3	0.5	224 ± 4.5	0.19 ± 0.02	−12.2 ± 1.8	-
S10	Tw60:Sp60:Chol 3.5:3.5:3	1	237 ± 6.9	0.26 ± 0.06	−11.7 ± 0.6	-
S11	Tw60:Sp60:Chol 3.5:3.5:3	2.5	250 ± 5.3	0.31 ± 0.05	−10.1 ± 1.4	-
S12	Tw60:Sp60:Chol 3.5:3.5:3	2.5	240 ± 3.3	0.38 ±0.03	−11.3 ± 2.1	89.3 ± 2.1
Niosomes modified with 3F-3K						
S13	Tw60:Sp60:Chol 3.5:3.5:3	0.5	218 ± 8.5	0.27 ± 0.02	−12.4 ± 0.4	-
S14	Tw60:Sp60:Chol 3.5:3.5:3	1	234 ± 5.7	0.31 ± 0.06	−11.3 ± 1.5	-
S15	Tw60:Sp60:Chol 3.5:3.5:3	2.5	240 ± 7.6	0.27 ± 0.02	−10.1 ± 1.7	-
S16	Tw60:Sp60:Chol 3.5:3.5:3	2.5	235 ± 5.6	0.36 ± 0.02	−11.1 ± 1.7	94.1 ± 2.8
Niosomes modified with 4F-3K						
S17	Tw60:Sp60:Chol 3.5:3.5:3	0.5	234 ± 8.5	0.41 ± 0.04	−10.2 ± 0.2	-
S18	Tw60:Sp60:Chol 3.5:3.5:3	1	223 ± 5.7	0.31 ± 0.05	−9.6 ± 0.1	-
S19	Tw60:Sp60:Chol 3.5:3.5:3	2.5	243 ± 7.6	0.36 ± 0.03	−9.5 ± 0.4	-
S20	Tw60:Sp60:Chol 3.5:3.5:3	2.5	238 ± 9.6	0.40 ± 0.05	−8.2 ± 0.3	90.1 ± 2.3

**Table 3:** Coefficient of determination (R^2^), release rate constant (K), release half time (t_1/2_), and diffusion exponent (n), after fitting of release profiles to different drug release kinetic models.

Kinetic Model	Zero Order		First Order		Higuchi		Korsmeyer–Peppas			
	R^2^	K_o_ (mg/mL)/h	R^2^	K_F_ (h^−1^)	R^2^	K_H_ (mg/mL)/h^0.5^	R^2^	K_KP_ (h^−n^)	n	t_1/2_ (h)
Tw60:Sp60:Chol non-modified niosomes (in PBS)	0.742	0.686	0.897	−0.0089	0.718	7.174	0.983	12.56	0.313	82.204
Tw60:Sp60:Chol non-modified niosomes (in PBS+ 20 albumin)	0.813	0.634	0.914	−0.008	0.712	7.01	0.989	17.61	0.23	81.58
Tw60:Sp60:Chol:3F-3K (in PBS)	0.540	0.657	0.873	−0.0066	0.594	6.001	0.985	11.24	0.290	171.6
Tw60:Sp60:Chol:3F-3K (in PBS + 20% albumin)	0.595	0.680	0.900	−0.0072	0.596	6.542	0.982	12.25	0.290	127.7

**Table 4:** Stability study of selected CBD-loaded niosomes after one-month storage at 4 ± 2 °C.

Sample		Size (nm)	PDI	ζ-Potential (mV)	EE (%)
(S5)	Initial	150 ± 1.1	0.35 ± 0.07	−10.3 ± 1.6	92.2 ± 2.1
	After 1 month storage	132 ± 2.8	0.41 ± 0.09	−10.6 ± 2.1	90.5 ± 4.1
(S16)	Initial	235 ± 5.6	0.36 ± 0.02	−11.1 ± 1.7	94.1 ± 2.8
	After 1 month storage	228 ±2.2	0.39 ± 0.05	−11.8 ± 1.2	93.8 ± 1.9

**Table 5:** IC_50_ values of free or niosomal CBD against human urothelial cancer and CTCL-derived tumor cells.

IC_50_ (μM ± SD) in Terms of CBD Concentration			
Cell Line Sample	T-24	MJ	HUT-78
CBD (as solution)	12.2 ± 2.1	58.6 ± 5.9	30.1 ± 5.9
Tw60:Sp60:Ch:3F-3K * (S16)	32.1 ± 6.3	74.5 ± 4.4	63.9 ± 1.8
Tw60:Sp60:Ch:4F-3K * (S20)	37.3 ± 5.2	74.2 ± 3.8	64.6 ± 3.7
Tw60:Sp60:Ch (N.M.) (S5)	58.9 ± 6.5	69.1 ± 6.2	86.2 ± 3.1

* In all copolymer-modified niosomes, the concentration of copolymer is 2.5 mol % from the total lipid (TL; TL = 30 µmol/mL). N.M.—CBD-loaded non-modified niosomes.

## Data Availability

Not applicable.

## Supplementary Materials

The following supporting information can be downloaded at: https://www.mdpi.com/article/10.3390/pharmaceutics15102414/s1, Section S1. Synthesis and characterization of monomers, polymer precursors, and linear and star-like copolymers: Scheme S1. Schematic presentation of the synthesis of mono-functional PEEGE bearing a hydroxyl end group (OH-(PO)_7_(EEGE)_23_-Cy) by ring-opening anionic polymerization of EEGE in bulk; Figure S1. SEC of OH-(PO)_7_(EEGE)_23_-Cy and HC≡C-(PO)_7_(EEGE)_23_-Cy (RI trace, THF). Figure S2. ^1^H-NMR spectra of HC≡C-(PO)_7_(EEGE)_23_-Cy, OH-(PO)_7_(EEGE)_23_-Cy in CDCl_3_, and OH-(PO)_7_(G)_23_-Cy in DMSO. Scheme S2. Schematic presentations of the synthesis of mono-alkyne functional macroreagent HC≡C-(PO)_7_(EEGE)_23_-Cy via esterification of OH-(PO)_7_(EEGE)_23_-Cy with 4-pentynoic acid. Scheme S3. Schematic presentation of the synthesis of azide-terminated poly(ε-caprolactone) R[(CL)_9_N_3_]_2_ macroreagent by a two-step procedure involving mesylation of the primary hydroxyl end groups of R[(CL)_9_OH]_2_ and reaction with NaN_3_. Figure S3. ^1^H NMR spectra of bifunctional R[(CL)_9_OH]_2_, R[(CL)_9_OSO_2_CH_3_]_2_, and R[(CL)_9_N_3_]_2_ in CDCl_3_ (600 MHz). Figure S4. FTIR spectra of R[(CL)_9_OH]_2_, R[(CL)_9_ OSO_2_CH_3_]_2_, and R[(CL)_9_N_3_]_2_. Scheme S4. Schematic presentation of the synthesis of azide-terminated poly(ε-caprolactone) R[(CL)_6_N_3_]_3_ macroreagent by a two-step procedure involving mesylation of the primary hydroxyl end groups of R[(CL)_6_OH]_3_ and reaction with NaN_3_. Figure S5. ^1^H NMR spectra of trifunctional R[(CL)_6_OH]_3_, R[(CL)_6_OSO_2_CH_3_]_3_, and R[(CL)_6_N_3_]_3_ in CDCl_3_ (600 MHz). Figure S6. FTIR spectra of trifunctional R[(CL)_6_OH]_3_, R[(CL)_6_OSO_2_CH_3_]_3_, and R[(CL)_6_N_3_]_3_. Scheme S5. Schematic presentation of the chain extension of tetrafunctional hydroxyl-terminated R[(CL)_5_OH]_4_. Figure S7. SEC chromatograms of R[(CL)_2_OH]_4_ (CAPA4101) and the product after chain extension R[(CL)_5_OH]_4_. RI trace, THF. Scheme S6. Schematic presentation of the synthesis of azide-terminated tetrafunctional poly(ε-caprolactone) R[(CL)_5_OH]_4_ macroreagent by a two-step procedure involving mesylation of the primary hydroxyl end groups of R[(CL)_5_OH]_4_ and reaction with NaN_3_. Figure S8. FTIR spectra of tetrafunctional R[(CL)_5_OH]_4_, R[(CL)_5_OSO_2_CH_3_]_4_, and R[(CL)_5_N_3_]_4_. Figure S9. ^1^H NMR spectra of tetrafunctional R[(CL)_5_OH]_4_, R[(CL)_5_OSO_2_CH_3_]_4_, and R[(CL)_5_N_3_]_4_ in CDCl_3_ (600 MHz). Figure S10. SEC chromatogram of R[(CL)_9_(PO)_7_(EEGE)_23_]_2_ (RI trace, THF). Figure S11. ^1^H NMR spectra of (a) R[(CL)_9_(PO)_7_(EEGE)_23_]_2_ in CDCl_3_ and (b) R[(CL)_9_(PO)_7_(G)_23_]_2_ in CD_3_OH (600 MHz). Figure S12. SEC chromatogram of R[(CL)_6_(PO)_7_(EEGE)_23_]_3_ (RI trace, THF). Figure S13. ^1^H NMR spectra of (a) R[(CL)_6_(PO)_7_(EEGE)_23_]_3_ in CDCl_3_ and (b) R[(CL)_6_(PO)_7_(G)_23_]_3_ in CD_3_OH (600 MHz). Figure S14. SEC chromatogram of R[(CL)_5_(PO)_7_(EEGE)_23_]_4_, (RI trace, THF). Figure S15. ^1^H NMR spectra of (a) R[(CL)_5_(PO)_7_(EEGE)_23_]_4_ in CDCl_3_ and (b) [(CL)_5_(PO)_7_(G)_23_]_4_ in CD_3_OH (600 MHz). Section S2. Size and size distribution from DLS and cryo-TEM of conventional niosomes and copolymer-modified emty or CBD-loaded niosomes; Figure S16. Intensity autocorrelation functions from DLS for Tw60:Sp60:Chol 3.5:3.5:3, modified with 2.5 mol % of 3F-3K at an angle of 173° and temperature of 25 °C. Red and green correspond to the first and second consecutive measurements, respectively; Figure S17. Size distribution from DLS of empty (a-c) or CBD-loaded (d) Tw60:Sp60:Chol (3.5:3.5:3) plain or polymer-modified niosomes; Figure S18. Size distribution from cryo-TEM of Tw60:Sp60:Chol (3.5:3.5:3) niosomes, modified with 2.5 mol % 4F-3K. Number of objects counted: 100. Vesicle size range: 50–660 nm. Average size: 263 nm. References [55,56] are cited in the supplementary materials.
