# Supplementary material for: Development, Characterization and Pharmacological Evaluation of Cannabidiol-Loaded Long Circulating Niosomes
Source: Pharmaceutics. 2023 Oct 3;15(10):2414. doi: 10.3390/pharmaceutics15102414 (PMC10609774; doi:10.3390/pharmaceutics15102414)
Supplement: Supplementary file 1 [file pharmaceutics-15-02414-s001.zip › pharmaceutics-2602579-supplementary-update.pdf]

Supporting information

Development, characterization and pharmacological evaluation of cannabidiol-loaded long circulating niosomes

Viliana Gugleva[1], Katerina Ahchiyska[2], Dilyana Georgieva[2], Rositsa Mihaylova[3], Spiro Konstantinov[3], Erik Dimitrov[4], Natalia Toncheva-Moncheva[4], Stanislav Rangelov[4], Aleksander Forys[5], Barbara Trzebicka [5] and Denitsa Momekova[2,*]

[1] Department of Pharmaceutical Technologies, Faculty of Pharmacy, Medical University of Varna "Prof. Dr. Paraskev Stoyanov", 84 Tsar Osvoboditel Str., 9000 Varna, Bulgaria; viliana.gugleva@mu-varna.bg

[2] Department of Pharmaceutical Technology and Biopharmaceutics, Faculty of Pharmacy, Medical University of Sofia, 2 Dunav Str., 1000 Sofia, Bulgaria; 105523@students.mu-sofia.bg, dgeorgieva@pharmfac.mu-sofia.bg and dmomekova@pharmfac.mu-sofia.bg

[3] Department of Pharmacology, Pharmacotherapy and Toxicology, Faculty of Pharmacy, Medical University of Sofia, 2 Dunav Str., 1000 Sofia, Bulgaria; rositsa.a.mihaylova@gmail.com and skonstantinov@pharmfac.mu-sofia.bg

[4] Institute of Polymers, Bulgarian Academy of Sciences, bl.103 Akad. G. Bonchev Str.,1113, Sofia, Bulgaria;e_dimitrov@polymer.bas.bg, ntoncheva@polymer.bas.bg and rangelov@polymer.bas.bg

[5] Centre of Polymer and Carbon Materials, Polish Academy of Sciences, ul. M. Curie-Skłodowskiej 34, Zabrze, Poland, aforys@cmpw-pan.edu.pl and btrzebicka@cmpw-pan.edu.pl

* Correspondence: dmomekova@pharmfac.mu-sofia.bg, Tel.: +359 2 9236528

Section S1. Synthesis and characterization of monomers, polymer precursors, and linear and star-like copolymers

Synthesis of ethoxyethyl glycidyl ether (EEGE): EEGE was synthesized following a procedure described elsewhere [55]. p-Toluenesulfonic acid (1 g, 5.80 mmol) was added portion-wise to a magnetically stirred mixture of 2,3-epoxypropanol (40.0 g, 0.54 mol) and ethyl vinyl ether (200 mL, 2.10 mol) maintaining the temperature below 40 °C. The reaction mixture was stirred for 3 h and then sat. aq. $NaHCO_3$ (100 mL) was added. The organic layer was separated, dried over anhydrous Na_2SO_4 and the excess ethyl vinyl ether was evaporated under reduced pressure. EEGE (b.p. 152-154 °C) was collected by vacuum distillation at 44 °C and ca. $1x10^3$ Pa as a colorless liquid. A fraction with purity exceeding 99 % (GC) was used.

Mono-hydroxyl terminated poly(ethoxyethyl glycidyl ether) OH-(PO)$_7$(EEGE)$_{23}$: 0.0561 g (0.0010 mol, 1 eq) of potassium hydroxide were added to 0.1201 g (0.0012 mol, 1.2 eq) cyclohexanol (Cy) under an argon atmosphere. After stirring for 60 °C for 1 h the reaction mixture was freeze-dried with 2 mL of dry benzene. Then, EEGE (8.0 g, 0.0547 mol, 55 eq) was added at portions of 2 g per 48 h. The polymerization was carried out at 65 ˚C for 8 days. Afterwards, the temperature was adjusted at 40 °C, the unreacted EEGE was removed under vacuum and propylene oxide (0.346 g, 0.0064 mol) was added to the reaction medium via a Hamilton syringe under argon. After additional 8 h, the reaction was terminated with methanolic water solution. The product was extracted three times with 50 mL of hexane/DMSO mixture. Evaporation of the solvent from the combined extracts gave 7.1 g of OH-(PO)$_7$(EEGE)$_{23}$, 84 % yield. The polymerization reaction is presented in Scheme S1. SEC and ^{1}H NMR spectrum are shown in Figures S1 and S2. ^{1}H-NMR ($CDCl_3$, δ ppm): PEEGE - 1.15 - 1.4 ppm (m, -CH(C$\underline{H}_3$)-O-CH$_2$-C$\underline{H}_3$) side chain); 3.25 – 4.05 ppm (m, -O-C$\underline{H}_2$-C$\underline{H}$(C$\underline{H}_2$-O)-O- main chain and -CH(CH$_3$)-O-C$\underline{H}_2$-CH$_3$ side chain); 4.68–4.76 ppm (t, C$\underline{H}$(CH$_3$)-O-CH$_2$-CH$_3$ side chain); 1.4– 2.0 ppm (m, C$\underline{H}_2$ from cyclohexanol end group); PPO - 1.15 - 1.3 ppm (m, -CH$_2$-CH(C$\underline{H}_3$)-O); 3.25 – 4.05 ppm (m, -C$\underline{H}_2$-C$\underline{H}$(CH$_3$)-O); M_n^{HNMR}=3,800 g.mol^{-1}, M_n^{SEC}=2,600 g.mol^{-1}, M_w/M_n=1.09.

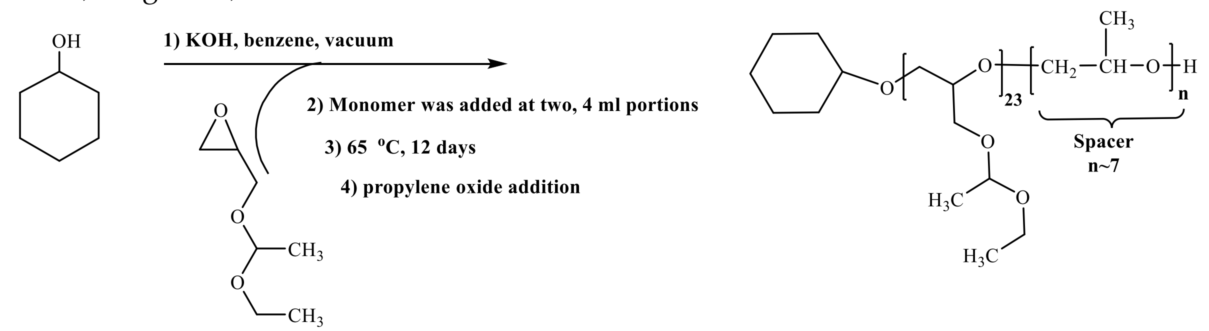

Scheme S1. Schematic presentation of the synthesis of mono-functional PEEGE bearing a hydroxyl end group (*OH-(PO)$_7$(EEGE)$_{23}$-Cy*) by ring-opening anionic polymerization of EEGE in bulk.

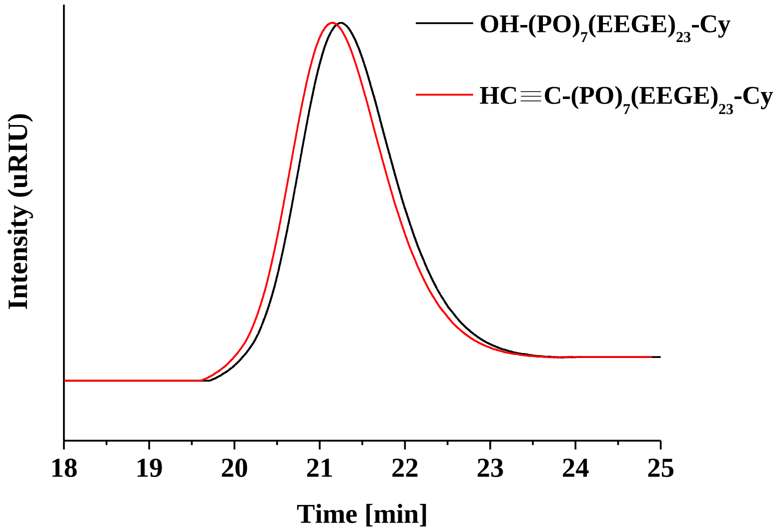

Figure S1. SEC of OH-(PO)$_7$(EEGE)$_{23}$-Cy and HC≡C-(PO)$_7$(EEGE)$_{23}$-Cy (RI trace, THF).

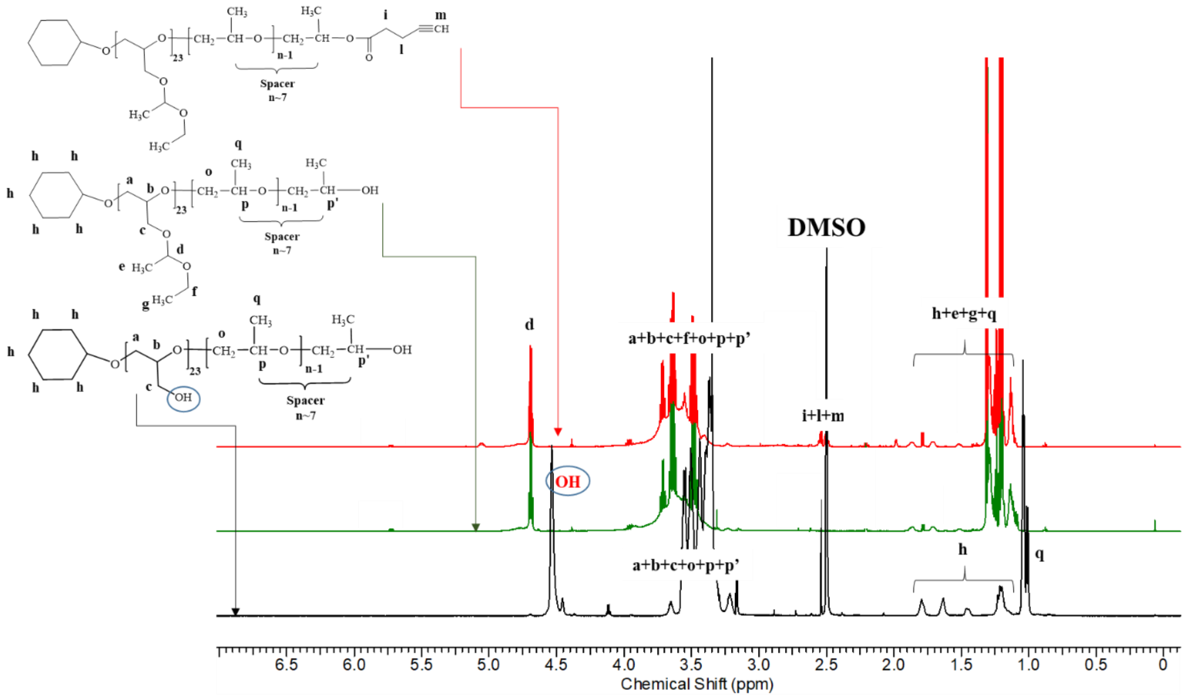

Figure S2. ^{1}H-NMR spectra of HC≡C-(PO)$_7$(EEGE)$_{23}$-Cy, OH-(PO)$_7$(EEGE)$_{23}$-Cy in $CDCl_3$ and OH-(PO)$_7$(G)$_{23}$-Cy in DMSO.

Mono-alkyne terminated poly(ethoxyethyl glycidyl ether) HC≡C-(PO)$_7$(EEGE)$_{23}$-Cy: In a typical example, OH-(PO)$_7$(EEGE)$_{23}$-Cy (1.8085 g, 0.4757 mmol) and 4-pentynoic acid (0.3733 g, 3.8063 mmol, 8 eq) were dried by azeotropic distillation with toluene. The dry polymer, 4-dimethylaminopyridine (0.1162 g, 0.9515 mmol, 2 eq), and (N-(3-dimethylaminopropyl)-N'-ethylcarbodiimide hydrochloride (0.3648 g, 1.9031 mmol, 4 eq) were dissolved in 5 mL of dry

chloroform in a 50 mL round-bottom flask. The solution was purged with argon for 30 min, and 4-pentynoic acid dissolved in 1 mL of dry chloroform was added dropwise. The reaction was carried out under argon at 40 °C for 120 h in the dark. The reaction mixture was filtered (0.45 μm PTFE filter) and dialyzed against a methanol/water mixture (10:1 v/v; membrane, MWCO 1000 Da) for 24 h in the dark. Then, methanol was evaporated by a rotary vacuum evaporator and HC≡C-$(PO)_7(EEGE)_{23}$-Cy was collected by freeze-drying. Yield: 1.55 g (86%); conversion: 99 %. The reaction pathway is presented in Scheme S2. SEC and ^{1}H NMR spectrum are shown in Figures S1 and S2.

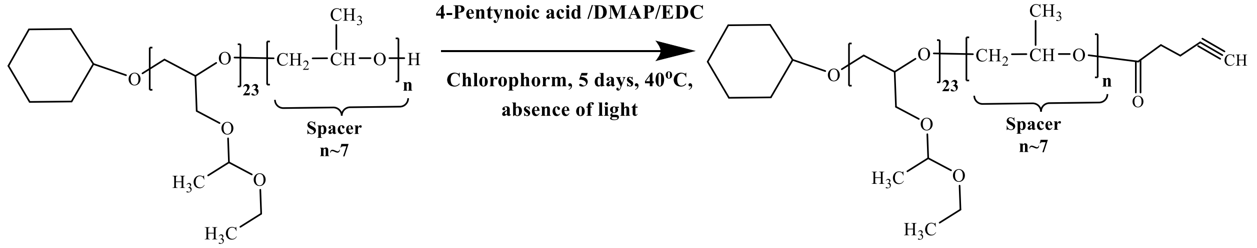

Scheme S2. Schematic presentations of the synthesis of mono-alkyne functional macroreagent HC≡C-$(PO)_7(EEGE)_{23}$-Cy via esterification of OH-$(PO)_7(EEGE)_{23}$-Cy with 4-pentynoic acid.

Cleavage of the protective ethoxyethyl groups: The deprotection procedure is based on the method, developed by Namboodiri et al. [34] and modified by Dimitrov et al. [35], using the Lewis acid $AlCl_3$. The amount of the latter was calculated according to the molar ratio $AlCl_3$:EEGE:CH_3OH=1:23:800. OH-$(PO)_7(EEGE)_{23}$-Cy (1.2 g, 23 eq) was dissolved in methanol (10.2 mL, 800 eq) at 40 °C and then $AlCl_3.6H_2O$ (0.07621 g, 1 eq) was added under stirring. The hydrolysis was conducted at the same temperature for 48 hours until complete disappearance of the methine proton signal at 4.75 ppm in the ^{1}H-NMR spectrum (Figure S2). The reaction mixture was filtered through Hyflo Super Cel® diatomaceous earth and then methanol was evaporated under reduced pressure. By this procedure the polymerization degree of the PPO spacer was precisely determined. The following composition of the precursor was determined: OH-$(PO)_7(EEGE)_{23}$-Cy.

Synthesis and characterization of azide terminated poly(ε-caprolactone) macroreagents $R[(CL)_9N_3]_2$, $R[(CL)_6N_3]_3$, and $R[(CL)_5N_3]_4$: Linear $R[(CL)_9N_3]_2$, and star-like 3-arm $R[(CL)_6N_3]_3$ and star-like 4-arm $R[(CL)_5N_3]_4$ macroreagents were synthesized following a procedure described elsewhere [33].

Hydroxyl terminated $R[(CL)_9OH]_2$ (CAPA 2205) (2 g, 1.00 mmol, 1 eq) was dissolved in dry CH_2Cl_2 (30 mL) in a 50 mL round-bottom flask under inert atmosphere. Then, DMAP (0.061 g, 0.5 mmol, 0.5 eq) and TEA (1.01 g, 10 mmol, 10 eq) were added to the reaction mixture. After stirring for 5 min at 0 °C, methanesulfonyl chloride (1.15 g, 10 mmol, 10 eq), dissolved in 10 mL of dry CH_2Cl_2, was added and the reaction mixture was stirred overnight at 25 °C. The product was added to water, forming a turbid solution and the polymer was extracted several times with CH_2Cl_2. The organic solution was dried using anhydrous $MgSO_4$ and filtered. The mesylated PCL was recovered after evaporation of CH_2Cl_2, precipitation in cold CH_3OH (-35 °C) and drying for 24 h in a vacuum oven at 25 °C. The synthesis and characterization by FTIR and ^{1}H NMR are

presented in Scheme S3 and Figures S3 and S4. Yield: 1.98 g (91 %); ^{1}H NMR ($CDCl_3$, δ ppm): 4.26 ppm (t, -CH_2-C$\underline{H}_2$-O-SO_2-), 4.05 (t, -C$\underline{H}_2$-O-CO)-), 2.3 (t, O(CO)-C$\underline{H}_2$-, 1.7-1.55 (m, O(CO)-CH_2-C$\underline{H}_2$-CH_2-C$\underline{H}_2$-CH_2-C(O)), 1.42-1.33 (m, O(CO)-CH_2-CH_2-C$\underline{H}_2$-CH_2-CH_2-C(O)). M_n^{SEC}= 3,200 g.mol^{-1}, M_w/M_n = 1.20.

The mesylated PCL (1.80 g, 0.85 mmol, 1 eq) was dissolved in dry N,N-dimethyl formamide (20 mL) under inert atmosphere in a 50 mL round-bottom flask and NaN_3 (1.6500 g, 24.6 mmol, 30 eq) was added. The reaction was carried out at 25 °C overnight. R[$(CL)_9$-N_3]$_2$ macroreagent was recovered by the same procedure as described above. The reaction scheme is shown in Scheme S3. ^{1}H NMR and FTIR spectra are shown in Figures S3 and S4. Yield: 1.78 g (96%); %. ^{1}H NMR ($CDCl_3$, δ ppm): 4.05 (t, -C$\underline{H}_2$-O-CO)-), 3.28 ppm (t, -CH_2-C$\underline{H}_2$-N_3), 2.3 (t, - O(CO)-C$\underline{H}_2$-, 1.7-1.55 (m, O(CO)-CH_2-C$\underline{H}_2$-CH_2-C$\underline{H}_2$-CH_2-C(O)), 1.42-1.33 (m, O(CO)-CH_2-CH_2-C$\underline{H}_2$-CH_2-CH_2-C(O); M_n^{SEC} = 3600 g.mol^{-1}, M_w/M_n = 1.20, conversion 99 %.

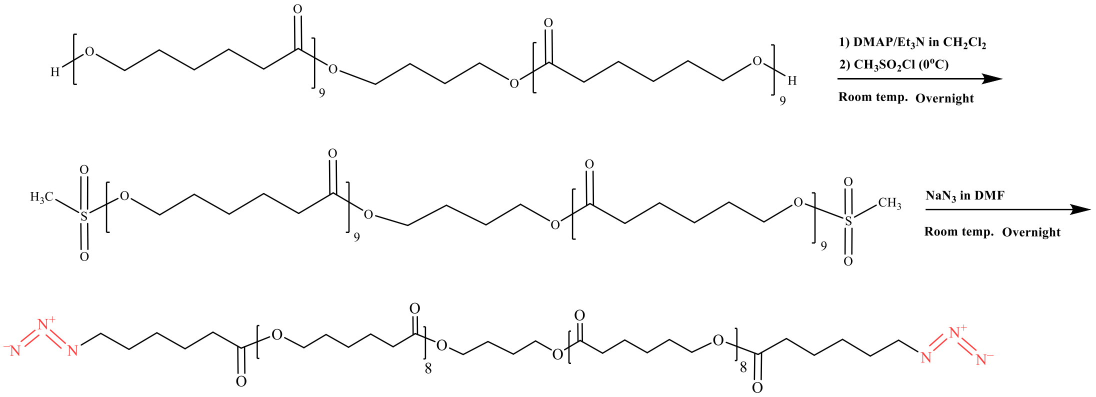

Scheme S3. Schematic presentation of the synthesis of azide terminated poly(ε-caprolactone) R[$(CL)_9N_3$]$_2$ macroreagent by a two-step procedure involving mesylation of the primary hydroxyl end groups of R[$(CL)_9OH$]$_2$ and reaction with NaN_3.

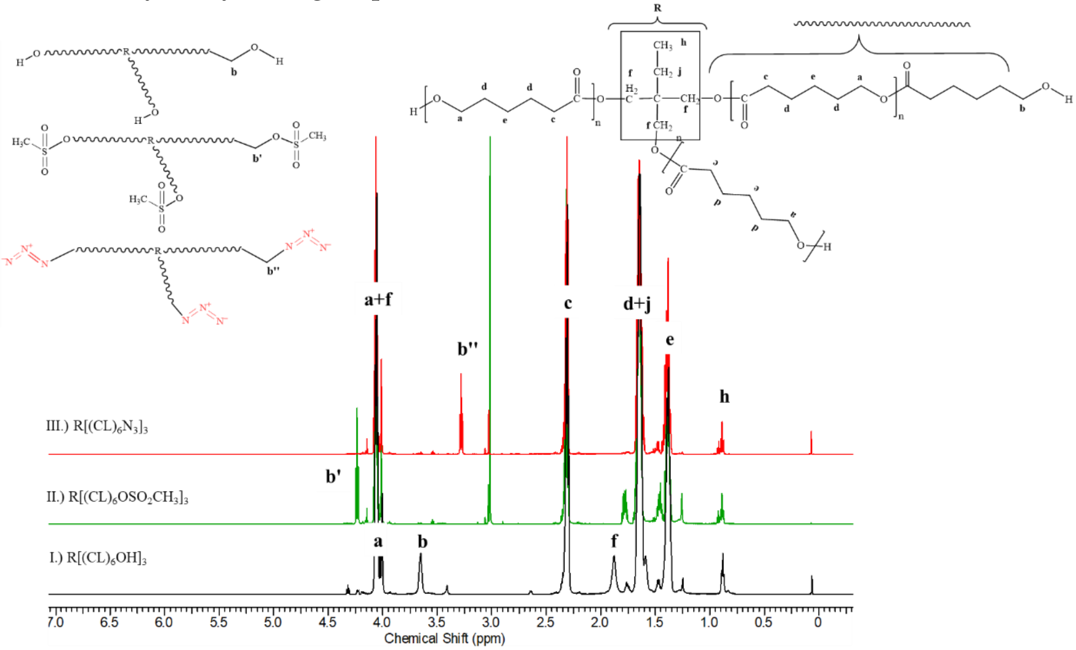

Figure S3. ^{1}H NMR spectra of bifunctional R[$(CL)_9OH$]$_2$, R[$(CL)_9OSO_2CH_3$]$_2$, and R[$(CL)_9N_3$]$_2$ in $CDCl_3$ (600 MHz).

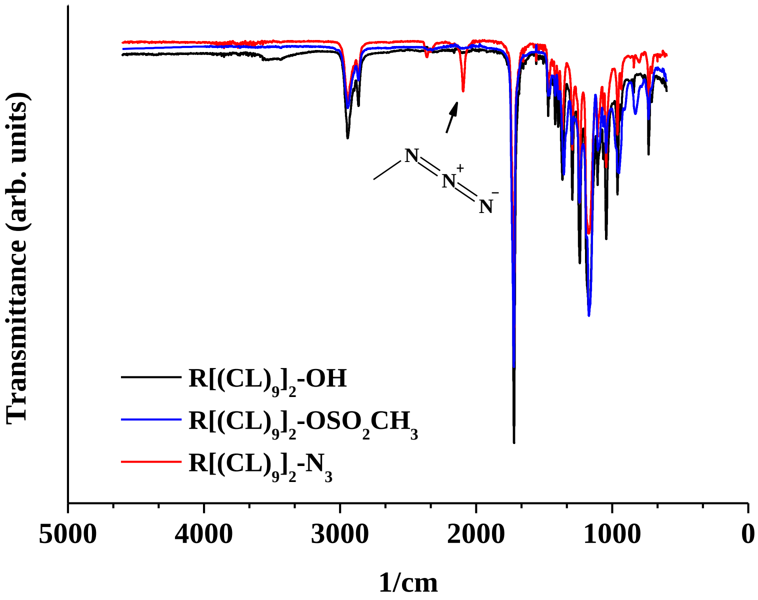

Figure S4. FTIR spectra of $R[(CL)_9OH]_2$, $R[(CL)_9\ OSO_2CH_3]_2$, and $R[(CL)_9N_3]_2$.

Hydroxyl terminated $R[(CL)_6OH]_3$ (CAPA 3201) (2 g, 1.00 mmol, 1eq) was dissolved in dry CH_2Cl_2 (30 mL) in a 50 mL round-bottom flask under inert atmosphere. Then, DMAP (0.092 g, 0.75 mmol, 0.75 eq) and TEA (1.51 g, 15 mmol, 15 eq) were added to the reaction mixture. After stirring for 5 min at 0 °C, methanesulfonyl chloride (1.15 g, 10 mmol, 10 eq), dissolved in 10 mL dry CH_2Cl_2, was added and the reaction mixture was stirred overnight at 25 °C. The product was added to water, forming a turbid solution and the polymer was extracted several times with CH_2Cl_2. The organic solution was dried using anhydrous $MgSO_4$ and filtered. The mesylated PCL was recovered after evaporation of CH_2Cl_2, precipitation in cold CH_3OH (-35 °C) and drying for 24 h in a vacuum oven at 25 °C. The synthesis and characterization by FTIR and ^{1}H NMR are presented in Scheme S4 and Figures S5 and S6. Yield: 1.74 g (87 %); ^{1}H NMR ($CDCl_3$, δ ppm): 4.24 ppm (t, -CH_2-C$\underline{H}_2$-O-SO_2-), 4.05 (t, -C$\underline{H}_2$-O-CO)-), 2.3 (t, O(CO)-C$\underline{H}_2$-, 1.7-1.55 (m, O(CO)-CH_2-C$\underline{H}_2$-CH_2-C$\underline{H}_2$-CH_2-C(O)), 1.42-1.33 (m, O(CO)-CH_2-CH_2-C$\underline{H}_2$-CH_2-CH_2-C(O)). M_n^{SEC}= 2,700 g.mol^{-1}, M_w/M_n = 1.29.

The mesylated PCL (1.80 g, 0.85 mmol, 1 eq) was dissolved in dry N,N-dimethyl formamide (20 mL) under inert atmosphere in a 50 mL round-bottom flask and NaN_3 (2.4800 g, 36.9 mmol, 45 eq) was added. The reaction was carried out at 25 °C overnight. $R[(CL)_6N_3]_3$ macroreagent was recovered by the same procedure as described above. The reaction scheme is presented in Scheme S4. ^{1}H NMR and FTIR spectra are shown in Figures S5 and S6. Yield: 1.62 g (90%); ^{1}H NMR ($CDCl_3$, δ ppm): 4.05 (t, -C$\underline{H}_2$-O-CO)-), 3.27 ppm (t, -CH_2-C$\underline{H}_2$-N_3), 2.3 (t, - O(CO)-C$\underline{H}_2$-, 1.7-1.55 (m, O(CO)-CH_2-C$\underline{H}_2$-CH_2-C$\underline{H}_2$-CH_2-C(O)), 1.42-1.33 (m, O(CO)-CH_2-CH_2-C$\underline{H}_2$-CH_2-CH_2-C(O); M_n^{SEC} = 2900 g.mol^{-1}, M_w/M_n = 1.28, conversion 99%.

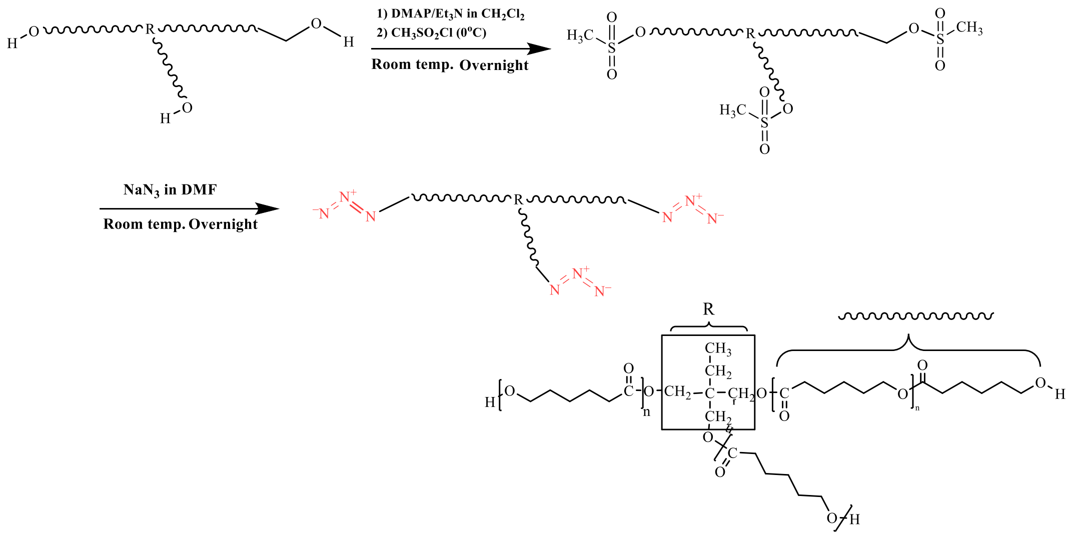

Scheme S4. Schematic presentation of the synthesis of azide terminated poly(ε-caprolactone) R[$(CL)_6N_3$]$_3$ macroreagent by a two-step procedure involving mesylation of the primary hydroxyl end groups of R[$(CL)_6OH$]$_3$ and reaction with NaN_3.

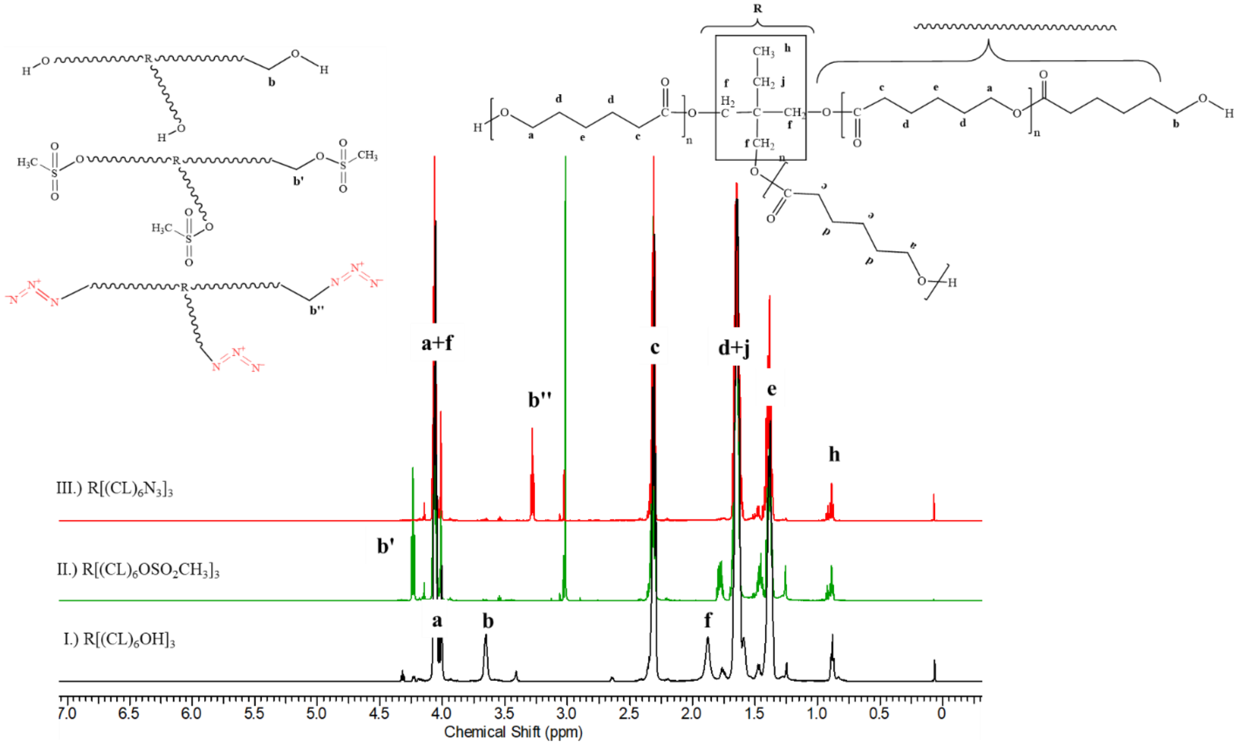

Figure S5. ^1H NMR spectra of trifunctional R[$(CL)_6OH$]$_3$, R[$(CL)_6OSO_2CH_3$]$_3$, and R[$(CL)_6N_3$]$_3$ in $CDCl_3$ (600 MHz).

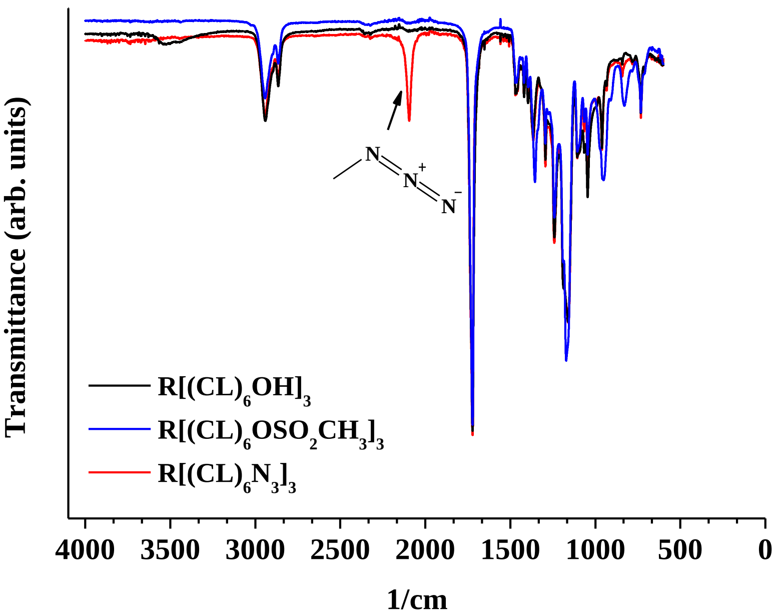

Figure S6. FTIR spectra of trifunctional $R[(CL)_6OH]_3$, $R[(CL)_6OSO_2CH_3]_3$, and $R[(CL)_6N_3]_3$.

Chain extension of tetrafunctional hydroxyl terminated $R[(CL)_2OH]_4$ *(CAPA4101) to* $R[(CL)_5OH]_4$ *in the presence of stannous octoate as initiator:* ε-caprolactone was polymerized following a procedure described elsewhere [56]. First, 3.65 g (3.65 mmol, 1 eq) of tetrafunctional hydroxyl terminated $R[(CL)_2OH]_4$ (CAPA 4101, M_n^{HNMR}=1,050 g.mol^{-1}, M_n^{SEC}=1,200 g.mol^{-1}, M_w/M_n = 1.13) were freeze-dried with 3 mL of dry benzene in a 50 ml round-bottom flask. Next, 5 g (44 mmol, 12 eq) of ε-caprolactone and 0.01 g (0.02 mmol, 0.007 eq) of stannous octoate were added under inert atmosphere. The polymerization was carried out in bulk at 130 °C for 3 h. The polymerization mixture was cooled and reaction was terminated with methanol (30 mL). Finally, 300 mL of distilled water were added and the resulted emulsion was centrifuged. The resulted polymer was recovered by freeze-drying. The extension reaction is schematically presented in Scheme S5. SEC curves of the initial and final products are shown in Figure S7. Yield: 6.58 g (94%); M_n^{HNMR}=2,200 g.mol^{-1}, M_n^{SEC}=1,700 g.mol^{-1}, M_w/M_n = 1.08.

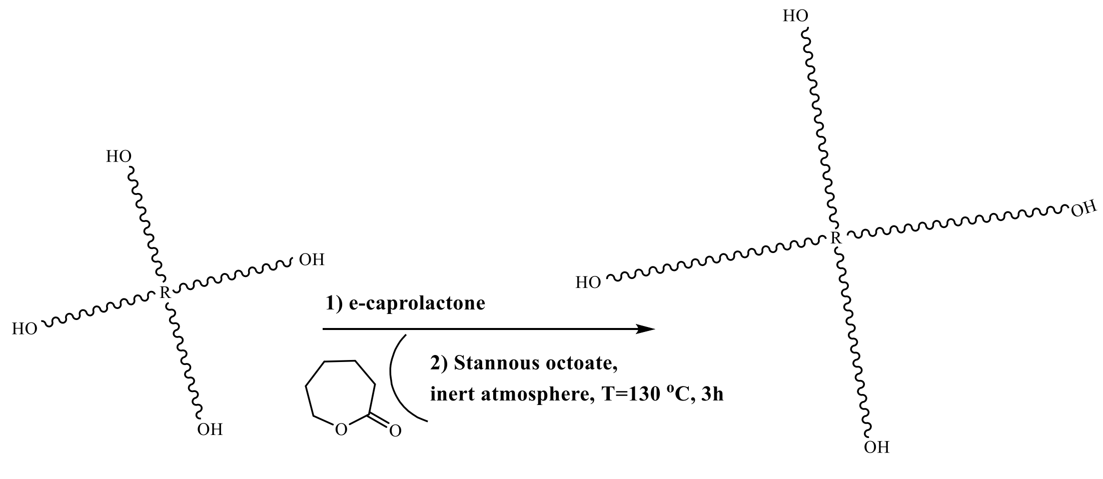

Hydroxyl terminated R[(CL)$_2$OH]$_4$ **Hydroxyl terminated R[(CL)$_5$OH]$_4$**

Scheme S5. Schematic presentation of the chain extension of tetrafunctional hydroxyl terminated R[(CL)$_5$OH]$_4$.

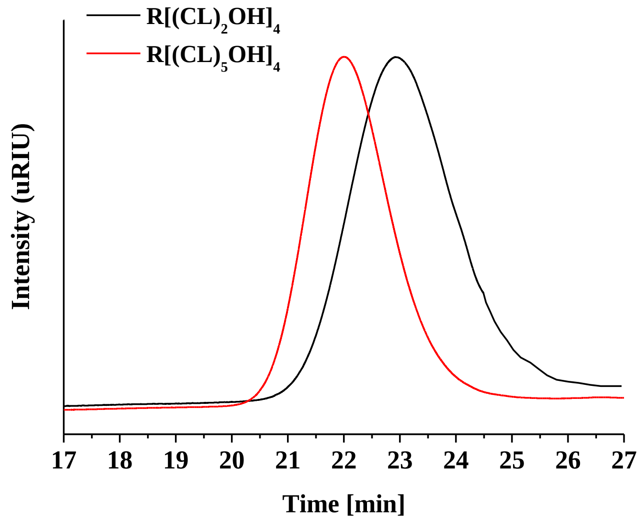

Figure S7. SEC chromatograms of R[(CL)$_2$OH]$_4$ (CAPA4101) and the product after chain extension R[(CL)$_5$OH]$_4$. RI trace, THF.

Tetrafunctional hydroxyl terminated R[(CL)$_5$OH]$_4$ (CAPA 4201) (2 g, 1.00 mmol, 1 eq) was dissolved in dry CH_2Cl_2 (30 mL) in a 50 mL round-bottom flask under inert atmosphere. Then, DMAP (0.123 g, 1 mmol, 1 eq) and TEA (2.01 g, 20 mmol, 20 eq) were added to the reaction mixture. After stirring for 5 min at 0 °C, methanesulfonyl chloride (1.53 g, 20 mmol, 20 eq), dissolved in 10 mL of dry CH_2Cl_2, was added and the reaction mixture was stirred overnight at 25 °C. The product was added to water, forming a turbid solution and the polymer was extracted several times with CH_2Cl_2. The organic solution was dried using anhydrous $MgSO_4$ and filtered. The mesylated PCL was recovered after evaporation of CH_2Cl_2, precipitation in cold CH_3OH (-35 °C) and drying for 24 h in a vacuum oven at 25 °C. The synthesis and characterization by FTIR and ^{1}H NMR are presented in Scheme S6 and Figures S8 and S9. Yield: 1.58 g (79 %); ^{1}H NMR

(CDCl$_3$, δ ppm): 4.24 ppm (t, $-CH_2-C\underline{H}_2-O-SO_2-$), 4.05 (t, $-C\underline{H}_2-O-CO)-$), 2.3 (t, $O(CO)-C\underline{H}_2-$, 1.7-1.55 (m, $O(CO)-CH_2-C\underline{H}_2-CH_2-C\underline{H}_2-CH_2-C(O)$), 1.42-1.33 (m, $O(CO)-CH_2-CH_2-C\underline{H}_2-CH_2-CH_2-C(O)$). M_n^{SEC}= 2,000 g.mol^{-1}, M_w/M_n = 1.09.

The mesylated PCL (1.80 g, 0.85 mmol, 1 eq) was dissolved in dry N,N-dimethyl formamide (20 mL) under inert atmosphere in a 50 mL round-bottom flask and NaN$_3$ (3.31 g, 49.2 mmol, 60 eq) was added. The reaction was carried out at 25 °C overnight. R[(CL)$_5$N$_3$]$_4$ macroreagent was recovered by the same procedure as described above. The reaction scheme is shown in Scheme S6. ^{1}H NMR and FTIR spectra are shown in Figures S8 and S9. Yield: 1.62 g (85 %); ^{1}H NMR (CDCl$_3$, δ ppm): 4.05 (t, $-C\underline{H}_2-O-CO)-$), 3.27 ppm (t, $-CH_2-C\underline{H}_2-N_3$), 2.3 (t, - $O(CO)-C\underline{H}_2-$, 1.7-1.55 (m, $O(CO)-CH_2-C\underline{H}_2-CH_2-C\underline{H}_2-CH_2-C(O)$), 1.42-1.33 (m, $O(CO)-CH_2-CH_2-C\underline{H}_2-CH_2-CH_2-C(O)$; M_n^{SEC} = 2,100 g.mol^{-1}, M_w/M_n = 1.08, conversion 99 %.

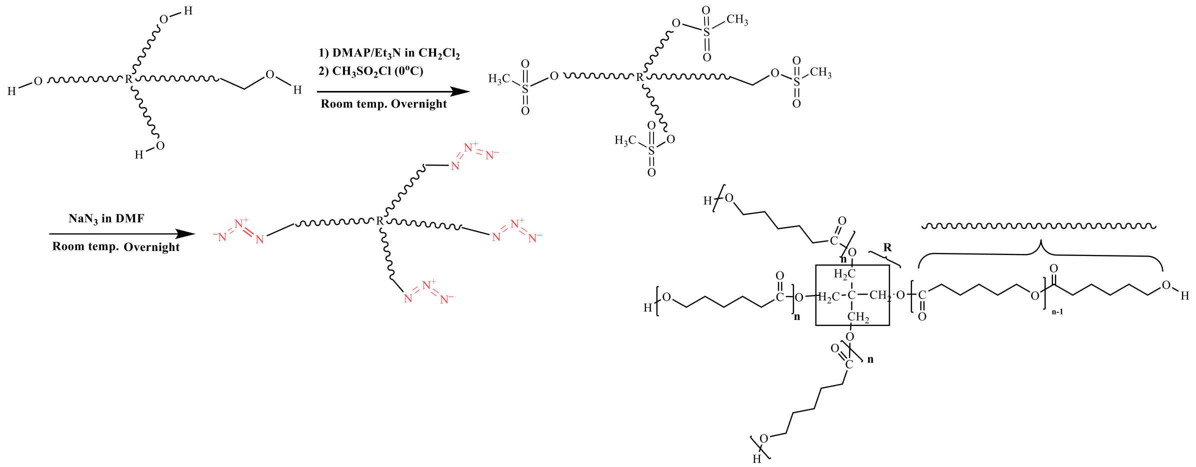

Scheme S6. Schematic presentation of the synthesis of azide terminated tetrafunctional poly(ε-caprolactone) R[(CL)$_5$OH]$_4$ macroreagent by a two-step procedure involving mesylation of the primary hydroxyl end groups of R[(CL)$_5$OH]$_4$ and reaction with NaN$_3$.

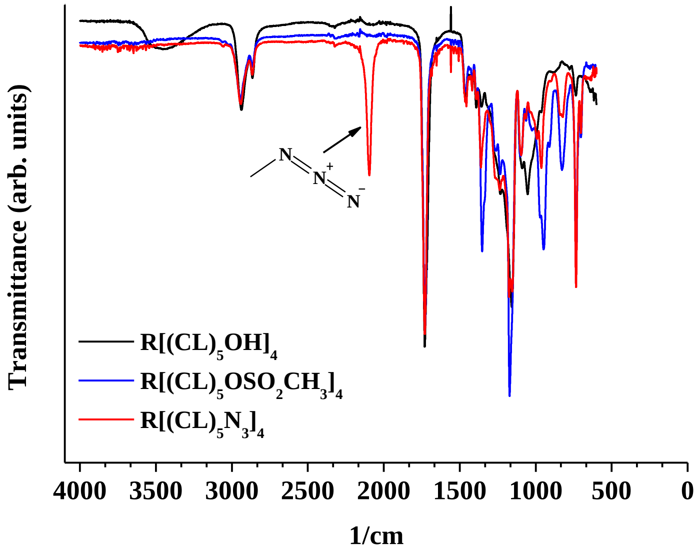

Figure S8. FTIR spectra of tetrafunctional R[(CL)$_5$OH]$_4$, R[(CL)$_5$OSO$_2$CH$_3$]$_4$, and R[(CL)$_5$N$_3$]$_4$.

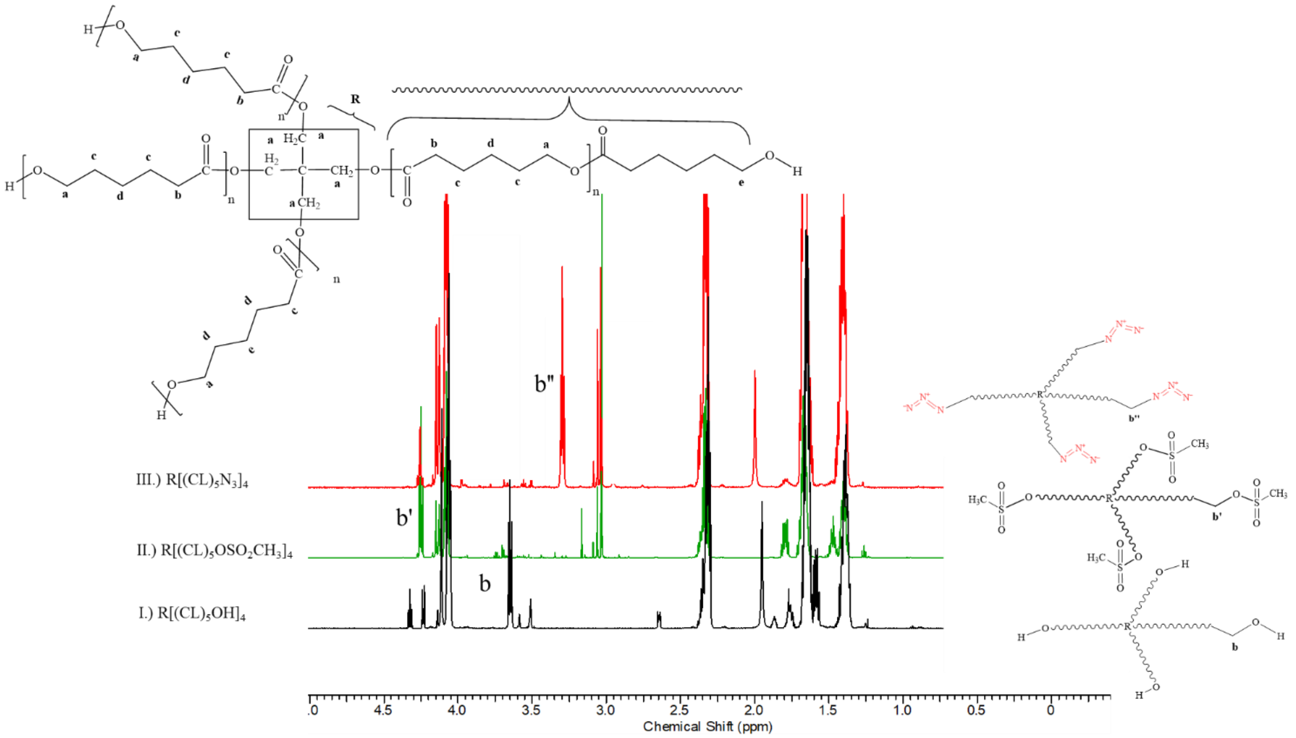

Figure S9. ^{1}H NMR spectra of tetrafunctional $R[(CL)_5OH]_4$, $R[(CL)_5OSO_2CH_3]_4$, and $R[(CL)_5N_3]_4$ in $CDCl_3$ (600 MHz).

Synthesis of poly(ethoxyethyl glycidyl ether)/poly(ε-caprolactone) linear $R[(CL)_9(PO)_7(EEGE)_{23}]_2$ and star-like $R[(CL)_6(PO)_7(EEGE)_{23}]_3$, $R[(CL)_5(PO)_7(EEGE)_{23}]_4$ copolymers by click coupling reaction.

$R[(CL)_9(PO)_7(EEGE)_{23}]_2$: $R[(CL)_9N_3]_2$ (0.257 g, 0.1285 mmol, 1 eq) and CuBr (0.368 g, 2.56 mmol, 20 eq) were placed in a 50 mL round-bottom flask under an argon atmosphere. Dry THF (3 mL) was added via a syringe, and the solution was purged with argon and stirred vigorously for 20 min. Monoalkyne-terminated HC≡C-$(PO)_7(EEGE)_{23}$ (0.9766 g, 0.2570 mmol, 2 eq) was dissolved in dry THF (2 mL) and added to the PCL solution along with PMDETA (0.6670 g, 3.85 mmol, 30 eq). The *click* coupling reaction was carried out at 30 °C for 24 h. The reaction mixture was cooled to RT, diluted with THF (30 mL), and filtered through a column filled with neutral alumina to remove copper complexes. The excess THF was evaporated, the crude product was dissolved in methanol (10 mL) and dialyzed against a methanol/water mixture (10:1 v/v, membrane, MWCO 8 kDa) for 72 h. The methanol was removed using a rotary vacuum evaporator, and the copolymer was recovered by freeze-drying. SEC chromatogram and ^{1}H NMR spectrum are shown in Figures S10 and S11.Yield: 0.6968 g (68%); M_n^{HNMR}=9,600 g.mol^{-1}, M_n^{SEC}=13,200 g.mol^{-1}, M_w/M_n = 1.20.

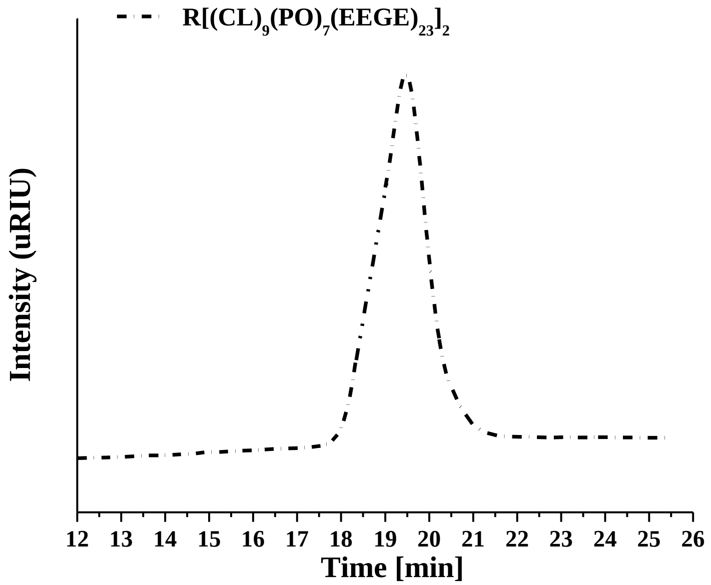

Figure S10. SEC chromatogram of R[$(CL)_9(PO)_7(EEGE)_{23}]_2$ (RI trace, THF).

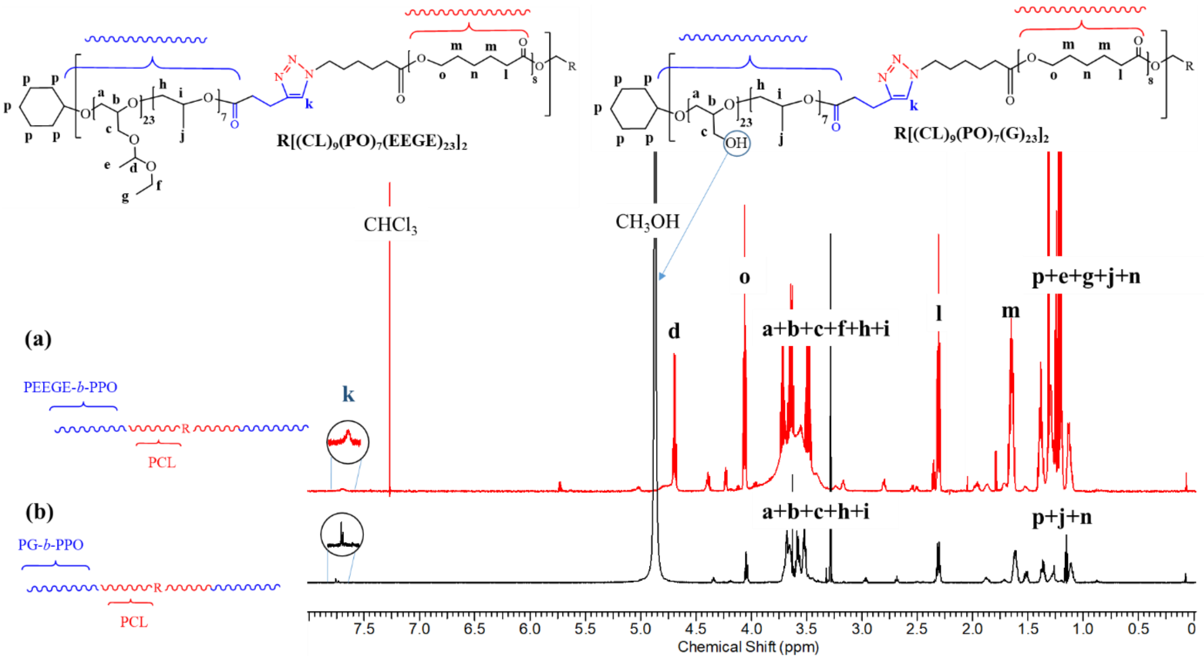

Figure S11. ^{1}H NMR spectra of (a) R[$(CL)_9(PO)_7(EEGE)_{23}]_2$ in $CDCl_3$ and (b) R[$(CL)_9(PO)_7(G)_{23}]_2$ in CD_3OH (600 MHz).

R[$(CL)_6(PO)_7(EEGE)_{23}]_3$: Azide terminated trifunctional R[$(CL)_6N_3]_3$ (0.2550 g, 0.1275 mmol, 1 eq) and CuBr (0.550 g, 3.83 mmol, 30 eq) were placed in a 50 mL round-bottom flask under an argon atmosphere. Dry THF (3 mL) was added via a syringe, and the solution was purged with argon and stirred vigorously for 20 min. Monoalkyne-terminated HC≡C-$(PO)_7(EEGE)_{23}$ (1.4554 g, 0.3830 mmol, 3 eq) was dissolved in dry THF (3 mL) and added to the PCL solution along with PMDETA (0.9960 g, 5.75 mmol, 40 eq). The *click* coupling reaction was carried out at 30 °C for 24 h. The

reaction mixture was cooled to RT, diluted with THF (30 mL), and filtered through a column filled with neutral alumina to remove copper complexes. The excess THF was evaporated, the crude product was dissolved in methanol (10 mL) and dialyzed against a methanol/water mixture (10:1 v/v, membrane, MWCO 8 kDa) for 72 h. The methanol was removed, using a rotary vacuum evaporator, and the copolymer was recovered by freeze-drying. SEC chromatogram and ^{1}H NMR spectrum are shown in Figures S12 and S13. Yield: 0.7915 g (56%); M_n^{HNMR}=13,400 g.mol^{-1}, M_n^{SEC}=17,700 g.mol^{-1}, M_w/M_n = 1.22.

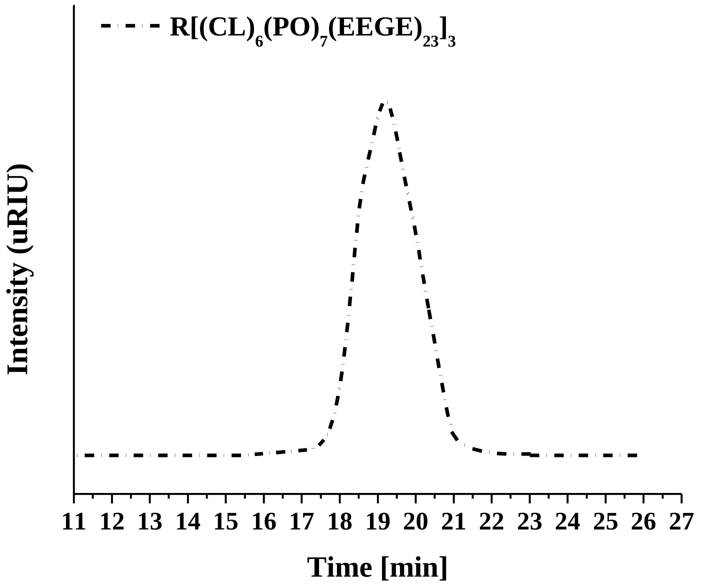

Figure S12. SEC chromatogram of R[(CL)$_6$(PO)$_7$(EEGE)$_{23}$]$_3$ (RI trace, THF).

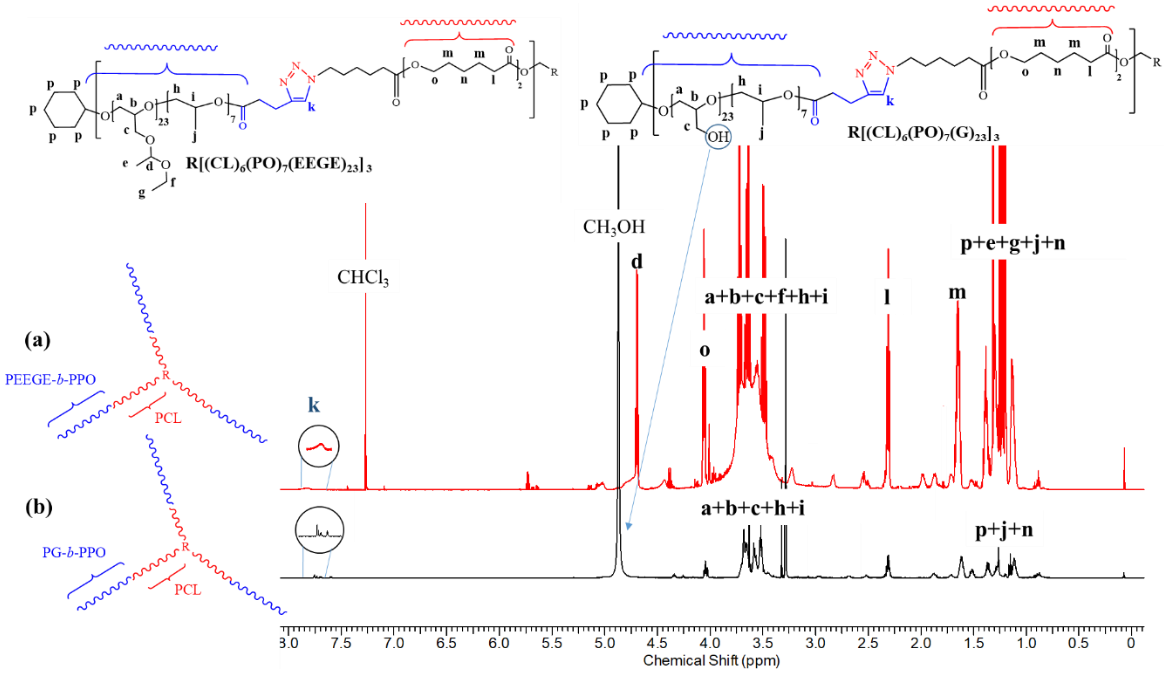

Figure S13. ^{1}H NMR spectra of (a) R[(CL)$_6$(PO)$_7$(EEGE)$_{23}$]$_3$ in $CDCl_3$ and (b) R[(CL)$_6$(PO)$_7$(G)$_{23}$]$_3$ in CD_3OH (600 MHz).

R[(CL)$_5$(PO)$_7$(EEGE)$_{23}$]$_4$: Azide terminated tetrafunctional [PCL$_5$N$_3$]$_4$ (0.2570 g, 0.1275 mmol, 1 eq) and CuBr (0.736 g, 5.13 mmol, 40 eq) were placed in a 50 mL round-bottom flask under an argon atmosphere. Dry THF (3 mL) was added via a syringe, and the solution was purged with argon and stirred vigorously for 20 min. Monoalkyne-terminated HC≡C-(PO)$_7$(EEGE)$_{23}$ (1.953 g, 0.514 mmol, 4 eq) was dissolved in dry THF (4 mL) and added to the PCL solution along with PMDETA (1.3300 g, 7.67 mmol, 60 eq). The *click* coupling reaction was carried out at 30 °C for 24 h. The reaction mixture was cooled to RT, diluted with THF (30 mL), and filtered through a column filled with neutral alumina to remove copper complexes. The excess THF was evaporated, the crude product was dissolved in methanol (10 mL) and dialyzed against a methanol/water mixture (10:1 v/v, membrane, MWCO 8 kDa) for 72 h. The methanol was removed, using a rotary vacuum evaporator, and the copolymer was recovered by freeze-drying. SEC chromatogram and ^{1}H NMR spectrum are shown in Figures S14 and S15. Yield: 0.667 g (37%); M_n^{HNMR}=17,200 g.mol^{-1}, M_n^{SEC}=14,100 g.mol^{-1}, M_w/M_n = 1.17.

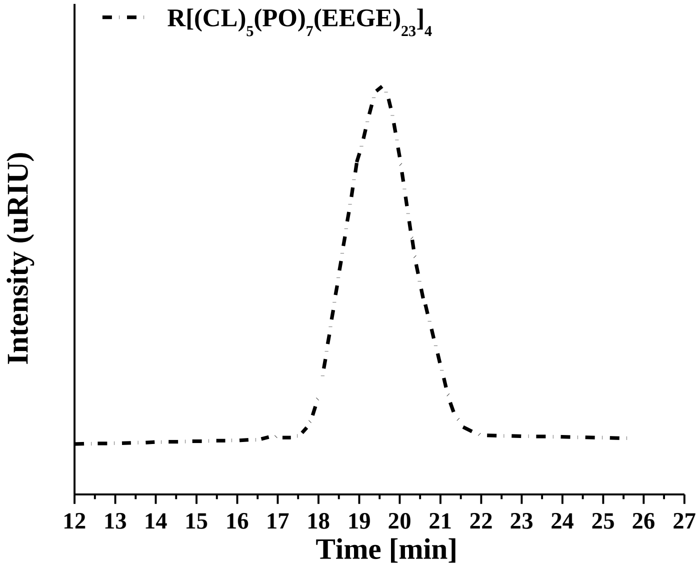

Figure S14. SEC chromatogram of R[(CL)$_5$(PO)$_7$(EEGE)$_{23}$]$_4$, (RI trace, THF).

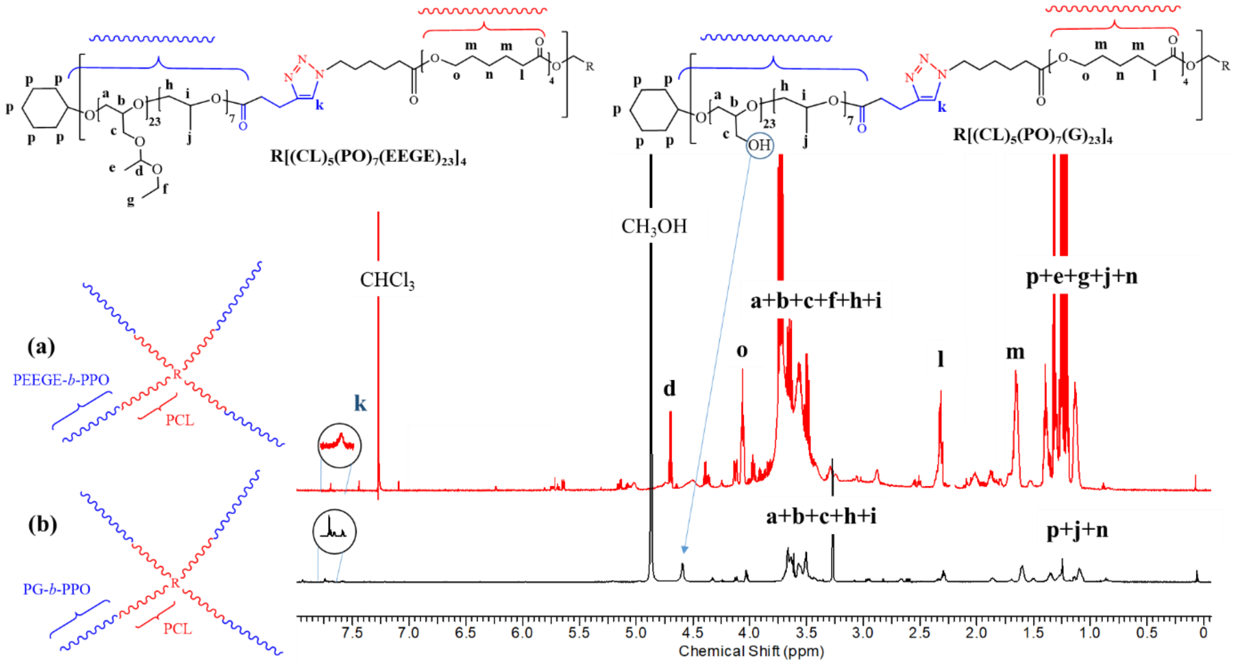

Figure S15. ^{1}H NMR spectra of (a) R[$(CL)_5(PO)_7(EEGE)_{23}]_4$ in $CDCl_3$ and (b) $[(CL)_5(PO)_7(G)_{23}]_4$ in CD_3OH (600 MHz).

Section S2. Size and size distribution from DLS and cryo-TEM of conventional niosomes, and copolymer modified empty or CBD loaded niosomes.

Generally, the intensity autocorrelation functions from DLS were single-exponential, corresponding to monomodal size distribution. The standard deviations (SD) of mean vesicle size were obtained from several (2-5, typically 3, not less than 2) consecutive measurements per sample. Given the apparent stability of niosomes, no dynamic changes in the sample occurred during the measurements that would lead to large SD. Figure S16 shows the raw correlation data for the sample Tw60:Sp60:Chol 3.5:3.5:3, modified with 2.5 mol % of 3F-3K (sample S15 from Table 2). The mean vesicle size was 240 ± 7.6 nm, determined from 245.5 nm and 235 nm from the first and second measurement, respectively.

The size distributions, numerically represented by PDI values in the 0.19 – 0.41 range (see Table 2 and Figure S17), may seem somewhat large but they are acceptable for drug delivery applications and consistent with the way of preparation of the niosomes – thin film hydration.

The cryo-TEM study corroborates the findings of the DLS investigation. The analysis of at least 100 objects per sample gave size and size distribution consistent with those obtained by DLS as exemplified with the formulation Tw60:Sp60:Chol 3.5:3.5:3, modified with 2.5 mol % of 4F-3K in Figure S18.

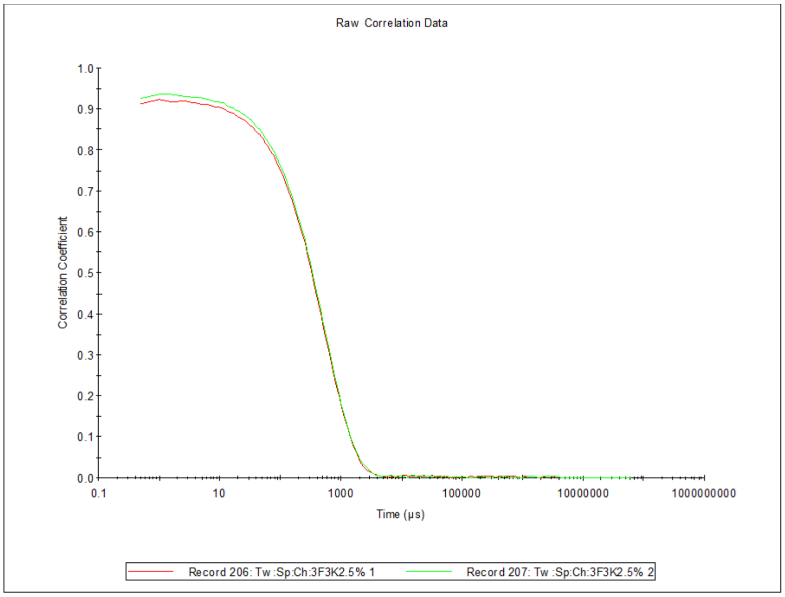

Figure S16. Intensity autocorrelation functions from DLS for Tw60:Sp60:Chol 3.5:3.5:3, modified with 2.5 mol % of 3F-3K at an angle of 173° and temperature of 25 °C. Red and green correspond to the first and second consecutive measurements, respectively.

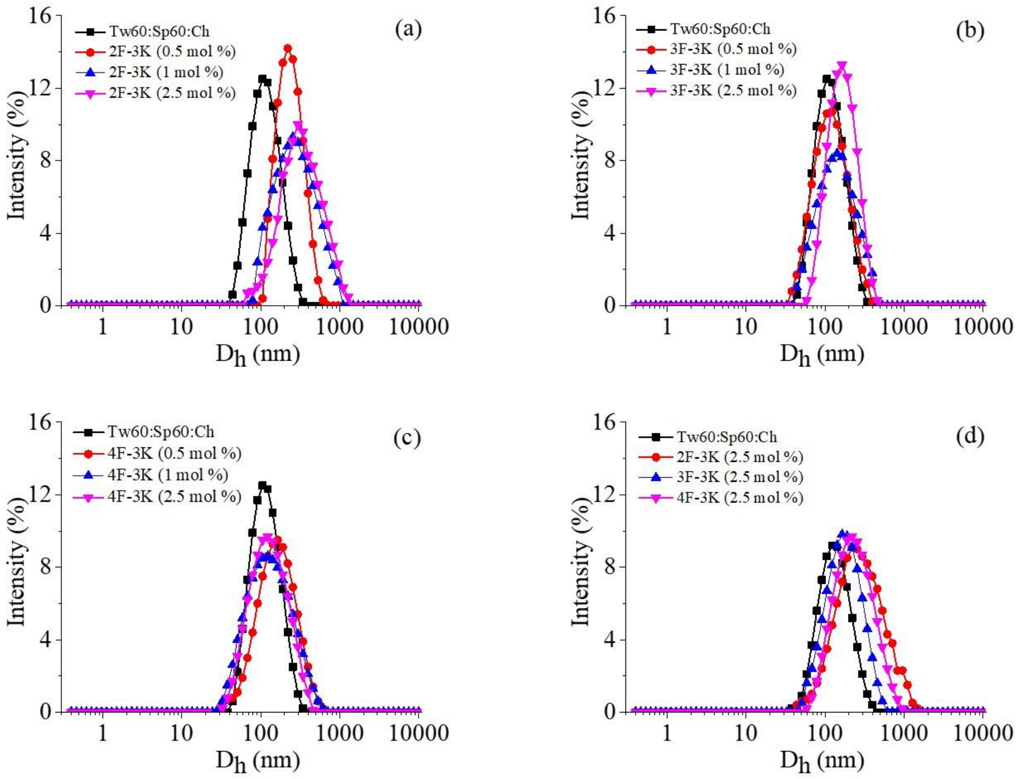

Figure S17. Size distribution from DLS of empty (a-c) or CBD loaded (d) Tw60:Sp60:Chol (3.5:3.5:3) plain or polymer modified niosomes.

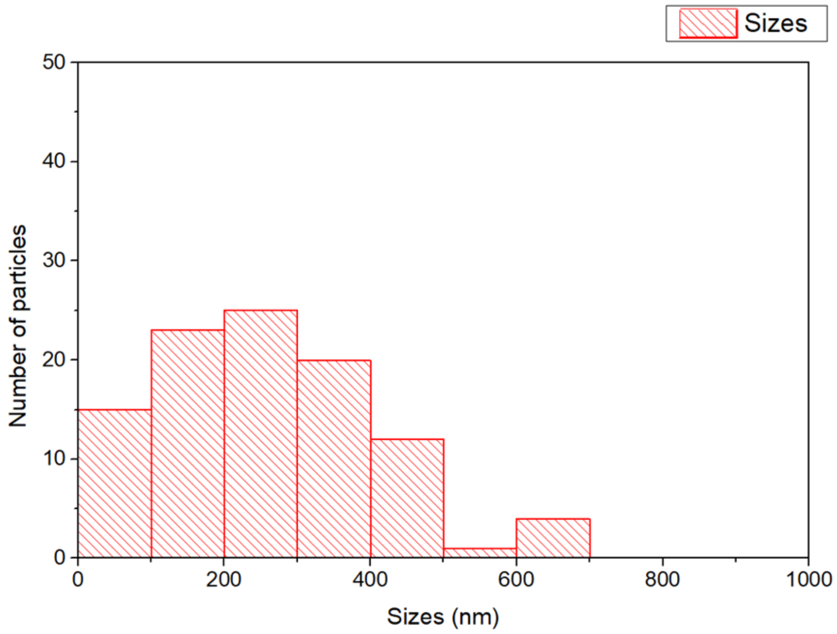

Figure S18. Size distribution from cryo-TEM of Tw60:Sp60:Chol (3.5:3.5:3) niosomes, modified with 2.5 mol % 4F-3K. Number of objects counted: 100. Vesicle size range: 50-660 nm. Average size: 263 nm.

References

[55] Fitton, A. O.; Hill, J.; Jane, D. E.; Millar, R. Synthesis of simple oxetanes carrying reactive 2-substituents. *Synthesis* **1987**, 1140–1142.

[56] Storey, R. F.; Sherman, J. W. Kinetics and Mechanism of the Stannous Octoate-Catalyzed Bulk Polymerization of ε-Caprolactone. *Macromolecules* **2002**, *35*, 1504-1512.
